# Molecular tools enabling pennycress (*Thlaspi arvense*) as a model plant and oilseed cash cover crop

**DOI:** 10.1111/pbi.13014

**Published:** 2018-10-25

**Authors:** Michaela McGinn, Winthrop B. Phippen, Ratan Chopra, Sunil Bansal, Brice A. Jarvis, Mary E. Phippen, Kevin M. Dorn, Maliheh Esfahanian, Tara J. Nazarenus, Edgar B. Cahoon, Timothy P. Durrett, M. David Marks, John C. Sedbrook

**Affiliations:** ^1^ School of Biological Sciences Illinois State University Normal IL USA; ^2^ School of Agriculture Western Illinois University Macomb IL USA; ^3^ Department of Plant Biology University of Minnesota Saint Paul MN USA; ^4^ Department of Biochemistry and Molecular Biophysics Kansas State University Manhattan KS USA; ^5^ Center for Plant Science Innovation and Department of Biochemistry University of Nebraska‐Lincoln Lincoln NE USA

**Keywords:** pennycress, genome, domestication, triacylglycerol, CRISPR, transformation

## Abstract

*Thlapsi arvense* L. (pennycress) is being developed as a profitable oilseed cover crop for the winter fallow period throughout the temperate regions of the world, controlling soil erosion and nutrients run‐off on otherwise barren farmland. We demonstrate that pennycress can serve as a user‐friendly model system akin to Arabidopsis that is well‐suited for both laboratory and field experimentation. We sequenced the diploid genome of the spring‐type Spring 32‐10 inbred line (1C DNA content of 539 Mb; 2*n* = 14), identifying variation that may explain phenotypic differences with winter‐type pennycress, as well as predominantly a one‐to‐one correspondence with Arabidopsis genes, which makes translational research straightforward. We developed an *Agrobacterium*‐mediated floral dip transformation method (0.5% transformation efficiency) and introduced CRISPR‐Cas9 constructs to produce indel mutations in the putative *FATTY ACID ELONGATION1* (*FAE1*) gene, thereby abolishing erucic acid production and creating an edible seed oil comparable to that of canola. We also stably transformed pennycress with the *Euonymus alatus* diacylglycerol acetyltransferase (*EaDAcT*) gene, producing low‐viscosity acetyl‐triacylglycerol‐containing seed oil suitable as a diesel‐engine drop‐in fuel. Adoption of pennycress as a model system will accelerate oilseed‐crop translational research and facilitate pennycress’ rapid domestication to meet the growing sustainable food and fuel demands.

## Introduction

Pennycress (*Thlapsi arvense* L., Field Pennycress) is an oilseed‐producing plant of the Brassicaceae family, closely related to the model *Arabidopsis thaliana* (Arabidopsis) and many agronomically important members including the oilseeds rapeseed (*Brassica rapa* and *Brassica napus* varieties), canola (rapeseed variants producing edible oil), carinata (*Brassica carinata*), camelina (*Camelina sativa*) and lepidium (*Lepidium campestre*) (Franzke *et al*., 2011; Warwick *et al*., 2010). Pennycress is being advanced as a new winter annual cash cover crop to be grown throughout the temperate regions of the world. For example, pennycress has a short enough life cycle to be double‐cropped between full‐season corn and soybeans on otherwise unused farmland throughout the 80 million‐acre U.S. Midwest Corn Belt (Fan *et al*., 2013; Hatfield, 2012; Moser *et al*., 2009b; Sedbrook *et al*., 2014).

Pennycress possesses a unique combination of attributes including extreme cold tolerance, over‐wintering growth habit and early‐season maturity (Isbell, 2008). As a winter cover, pennycress provides important ecosystem services such as reductions in soil erosion and nutrients leaching, spring weed suppression, habitat for insects and an early‐season food source for pollinators (Eberle *et al*., 2015; Groeneveld and Klein, 2015; Johnson *et al*., 2015; Thom *et al*., 2018; Thomas *et al*., 2017).

Pennycress seeds weigh 0.6–1.3 mg each and contain 27%–39% oil and about 19% protein (dry weight basis) (Hojilla‐Evangelista *et al*., 2013; Moser *et al*., 2009a; Figures S1 and S2). Wild pennycress stands can grow to a density equivalent to the production of 2000 lbs of seed per acre (2200 kg/hectare) (Mitich, 1996), which equates to ~85 gallons of oil and ~1300 pounds of press cake meal per acre (790 litres/hectare and 1460 kg/hectare respectively). Both the oil and meal have potential economic value, which will help promote the adoption of pennycress as a new eco‐friendly cash crop that minimally competes for land with existing cash crops.

Existing cultivars of pennycress have only been removed from the wild for a few generations and still harbour many weed traits. Traits requiring domestication include reduced seed dormancy to improve germination and stand establishment (Gesch *et al*., 2016), reduced pod shatter to limit pre‐harvest seed losses, reduced seed glucosinolate and seed coat fibre content to improve the nutritional value and palatability of the meal, and reduced seed oil erucic acid content to produce an edible oil comparable to canola (Doebley *et al*., 2006; Sedbrook *et al*., 2014; Vaughan *et al*., 2007). Importantly, most of the genes that control these traits have already been identified through decades of research in Arabidopsis and crop plants (Provart *et al*., 2016).

Pennycress has a diploid genome consisting of seven chromosomes (2*n* = 14) and 539 Mb of DNA. To begin to capitalise on the vast store of information derived from Arabidopsis research, a transcriptome and draft genome for pennycress were generated for the winter annual line MN106 (Dorn *et al*., 2013, 2015). The draft captured most of the gene space in pennycress and identified over 27 000 candidate orthologues to Arabidopsis genes, including most of the key genes controlling the needed domestication traits described above. The draft genome also confirmed the close relationship of pennycress to the halophytic model *Eutrema salsugineum* (Eutrema) (Yang *et al*., 2013). Pennycress has undergone whole‐genome duplication similarly to that of Arabidopsis, which is in line with genome comparisons between Eutrema and Arabidopsis (Dorn *et al*., 2015). As a result, there is mostly a one‐to‐one correspondence between Arabidopsis genes and candidate pennycress orthologues. About 86% of pennycress genes have highly similar homologues in Arabidopsis. This is important because, in Arabidopsis, recessive mutations in many regulatory genes elicit the key domestication traits that are needed to make pennycress a new crop species. Thus, we can predict that similar mutations and traits can be created in pennycress.

The need to generate and identify mutations in pennycress coincides with recent advances in molecular techniques that facilitate site‐directed mutagenesis. Especially relevant is CRISPR gene editing methodology, which can be used to create small deletions and insertions as well as base substitutions in any chosen gene (Fauser *et al*., 2014; Komor *et al*., 2016; Scheben *et al*., 2017). This technology requires the ability to transform the target plants with gene constructs that mediate the generation of these mutations. Therefore, we began a programme to develop transformation techniques for pennycress. To speed this process, we developed and employed an inbred line named ‘Spring 32‐10’, which has low seed dormancy and does not require cold treatment to flower (vernalisation).

We found that Spring 32 inbred lines as well as other pennycress spring‐ and winter‐annual varieties could be transformed using an *Agrobacterium*‐mediated floral dip protocol identical to that used for Arabidopsis except for a required vacuum infiltration step. This has allowed the use of CRISPR‐Cas9‐based methodology to create deletion and insertion (indel) mutations in the pennycress *FAE1* gene resulting in the abolishment of erucic acid in the seed oil. This change (seed oil erucic acid content below 2%) along with a genetic change(s) to reduce seed glucosinolate levels below 30 micromoles will make pennycress seed oil and meal edible and highly palatable and should allow for regulatory approval for food and feed applications. In addition, as a proof of concept, pennycress was transformed with the *Euonymus alatus* diacylglycerol acetyltransferase (*EaDAcT*) gene to create a novel oil with low‐viscosity properties that can be used as a drop‐in fuel in diesel engines.

Given that pennycress can be easily grown in the laboratory as well as used for field experimentation, has a relatively small low‐redundant diploid genome similar to that of Arabidopsis, is closely related to other oilseed crops including canola, and can be readily transformed and genetically improved, a case can be made that pennycress is an excellent choice as a new model plant system that itself can be rapidly domesticated into a profitable oilseed crop.

## Results

### Pennycress cultivar Spring 32 is well‐suited for both laboratory and field experimentation

In the late 2000s, we initiated breeding programmes aimed at domesticating pennycress as a winter annual oilseed cover crop. As part of those efforts, seeds from nearly 200 pennycress populations were collected from across the U.S. and evaluated for a variety of agronomic traits including winter versus spring type, seed oil quantity and composition, time to flowering and seed size and yield. Seeds of 33 pennycress accessions, collected from around the world and available from the USDA‐ARS Germplasm Resources Information Network (GRIN), were also evaluated. Representative seed oil composition and seed weight data for a portion of those collections are listed in Figures S1 and S2. While genotypic/phenotypic variation exists allowing success in breeding efforts for some traits including yield, many traits did not exhibit enough variation to allow for the attainment of domestication targets strictly through breeding. For example, to meet food regulatory requirements, pennycress seed oil must have an erucic acid (C22:1) content near zero, yet the lowest naturally occurring amount of erucic acid identified was ~27% (Figure S1). Therefore, to attain traits like low erucic acid, a CRISPR‐Cas9 mutagenesis approach was developed (detailed below).

In the laboratory, we found that vernalisation of winter‐type pennycress isolates such as MN106 (source of the draft genome; original seed collected near Coates, MN (Dorn *et al*., 2013, 2015)), Beecher (collected near Hanna City, IL), and Elizabeth (a low seed dormancy isolate from the original Beecher population; Isbell *et al*., 2017) could be consistently induced with a 21‐day incubation at 4 °C, which is in line with previous reports for pennycress and other Brassicaceae (Best and Mc Intyre, 1972; Nordborg and Bergelson, 1999). This 21‐day 4 °C vernalisation treatment worked on plants growing on agar media or in soil, from young seedling stage to multi‐leaves rosette stage. Imbibing seeds in water and then leaving them damp in microfuge tubes at 4 °C in a refrigerator was also found to be an effective vernalisation treatment. Flowering could also be induced with varying success by spraying rosettes with 0.01 mm giberrellin A4 + A7 (GA_4+7_; see Materials and Methods).

**Figure 1:**
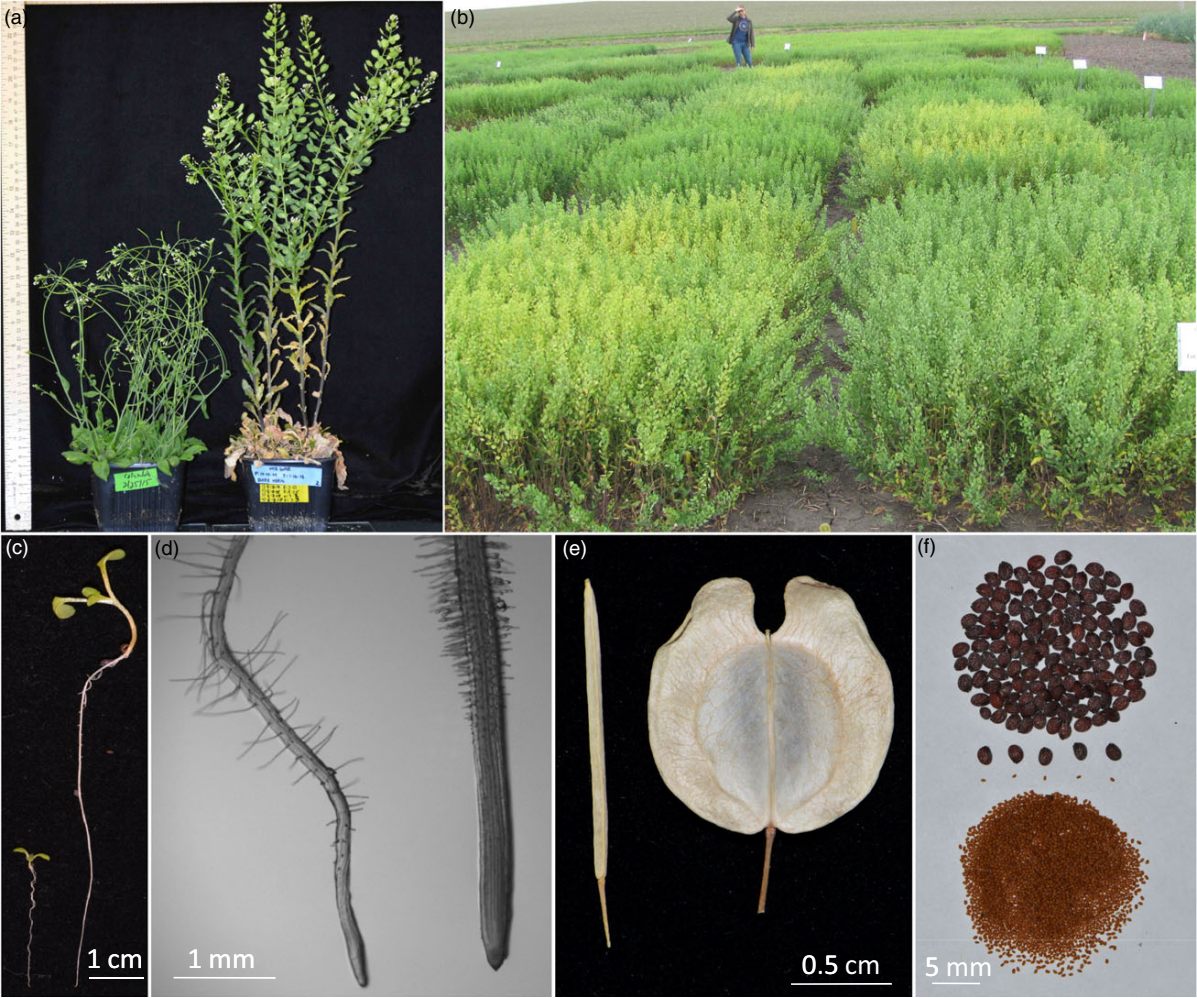
Pennycress (*Thlaspi arvense*) compared to Arabidopsis (*Arabidopsis thaliana*). (a) Arabidopsis (left) and pennycress (right) plants grown in the same soil mixture side by side in the same growth chamber. (b) Pennycress field plots (front left plot is cultivar Spring 32). (c) 7‐day‐old seedlings grown on vertically oriented agar medium. (d) Microscopic images of seedling roots grown on vertically oriented agar medium. (e) Saliques of senesced plants. (f) Seeds of pennycress (top) and Arabidopsis (bottom). In panels (a), (c), (d) and (e), Arabidopsis is positioned to the left of pennycress.

Since laboratory research using winter annual pennycress plants is slowed by the 21‐day vernalisation requirement, we developed inbred spring annual lines from a population named Spring 32. Spring‐type plants, by definition, do not require cold treatment to flower. The Spring 32 population was originally collected near Bozeman, MT and grown in two field seasons (September through May) near Macomb, IL (Figure 1), after which individual plants were carried forward by self‐pollination and single seed descent for ten generations under laboratory conditions (see Materials and Methods). For each generation, the best‐germinating seed and best‐growing plant was selected, culminating in inbred line Spring 32‐10.

Spring 32 and derived inbred plants were found to grow consistently well both in laboratory and field settings (Figure 1). In the laboratory, Spring 32 as well as other pennycress isolates could be cultivated using the same techniques used for Arabidopsis, including being grown side by side in the same soil mixes and in the same growth‐chamber conditions. We found that Spring 32 plants, when grown in potting soil with no nitrogen added, produced mature seeds in as little as 9 weeks (Figure S3). Increasing amounts of nitrogen in the form of prilled urea as a soil amendment resulted in later flowering and senescence, more axillary branches and higher seed yields (Table 1 and Figure S3).

**Table 1:** Mean values of pennycress plant and seed data obtained from five nitrogen fertilisation treatments using spring‐type ‘Spring 32’ pennycress

Nitrogen rate^a^	Plant height (cm)	Height of first pod (cm)	Number of pods on leader	Total pods on a plant	Number of aborted fruits on leader	Number of branches	Number of axillary branches	1000 seed weight (g)	Seed weight per plant (g)	Biomass (g)	Harvest index (%)
0	41 a	21 a	45	90 a	10 a	7 a	0 a	1.29 a	0.8 a	0.46 a	63 a
25	44 a	21 a	46	96 a	14 ab	4 ab	0 a	1.17 ab	0.9 ab	0.72 a	54 a
50	38 b	17 b	42	126 ab	17 b	3 b	2 ab	1.10 ab	1.2 b	1.04 a	52 ab
75	47 a	21 ab	45	181 b	22 b	5 ab	2 ab	1.14 ab	1.6 c	1.82 b	46 ab
100	41 ab	15 b	39	178 b	20 b	6 ab	2 ab	1.11 b	1.7 c	2.02 b	44 b
125	39 ab	18 ab	41	228 b	21 b	4 ab	5 b	1.12 ab	2.1 c	2.64 c	42 b

Within columns, means followed by the same letters are not significantly different at 0.05 probability level. Columns with no letters are not significant.

a *N* = nitrogen rate (lbs./acre), 50 lbs N/acre equivalent to 0.063 g of 46% prilled urea pellets per 7.5 cm square pot.

While the seeds of Spring 32 and many of the other pennycress isolates we tested exhibited relatively low seed dormancy under laboratory growth conditions (e.g. could be planted immediately upon harvest), some pennycress varieties including the winter annual accession Beecher displayed primary seed dormancy especially upon harvest, requiring either a brief incubation (e.g. 0.5 h) in 0.01 mm giberrellin A4 + A7 (GA_4+7_) to break seed dormancy or after‐ripening for a few months at room temperature (Sedbrook *et al*., 2014). Interestingly, incubation with the commonly used GA_3_ was ineffective at breaking pennycress seed dormancy.

### Whole‐genome sequence analysis of inbred line Spring 32‐10

Purified genomic DNA from six Spring 32‐10 inbred‐line plants was pooled and sequenced using the Illumina HiSeq 2500 platform. Enough data were collected to predict 36X coverage. The sequence reads were re‐mapped to *Thlaspi* version 1, which was generated from the sequencing of the winter annual line MN106 (Dorn *et al*., 2015), using BWA tools. These efforts resulted in the annotation of 27 390 predicted genes in the Spring 32‐10 genome (Table S1). The haplotype caller from GATK discovered 409 743 variants in the Spring 32‐10 genome compared to the MN106 draft genome, of which 360 186 were SNPs and 49 557 were INDELs. The close evolutionary relationship between the *Eutrema salsugineum* (Eutrema) and *Thlaspi arvense* (pennycress) genomes (Esmailbegi *et al*., 2018; Franzke *et al*., 2011) including the same number of chromosomes (*n* = 7) allowed us to demonstrate the distribution of SNPs identified in the pennycress Spring 32‐10 inbred line.

**Figure 2:**
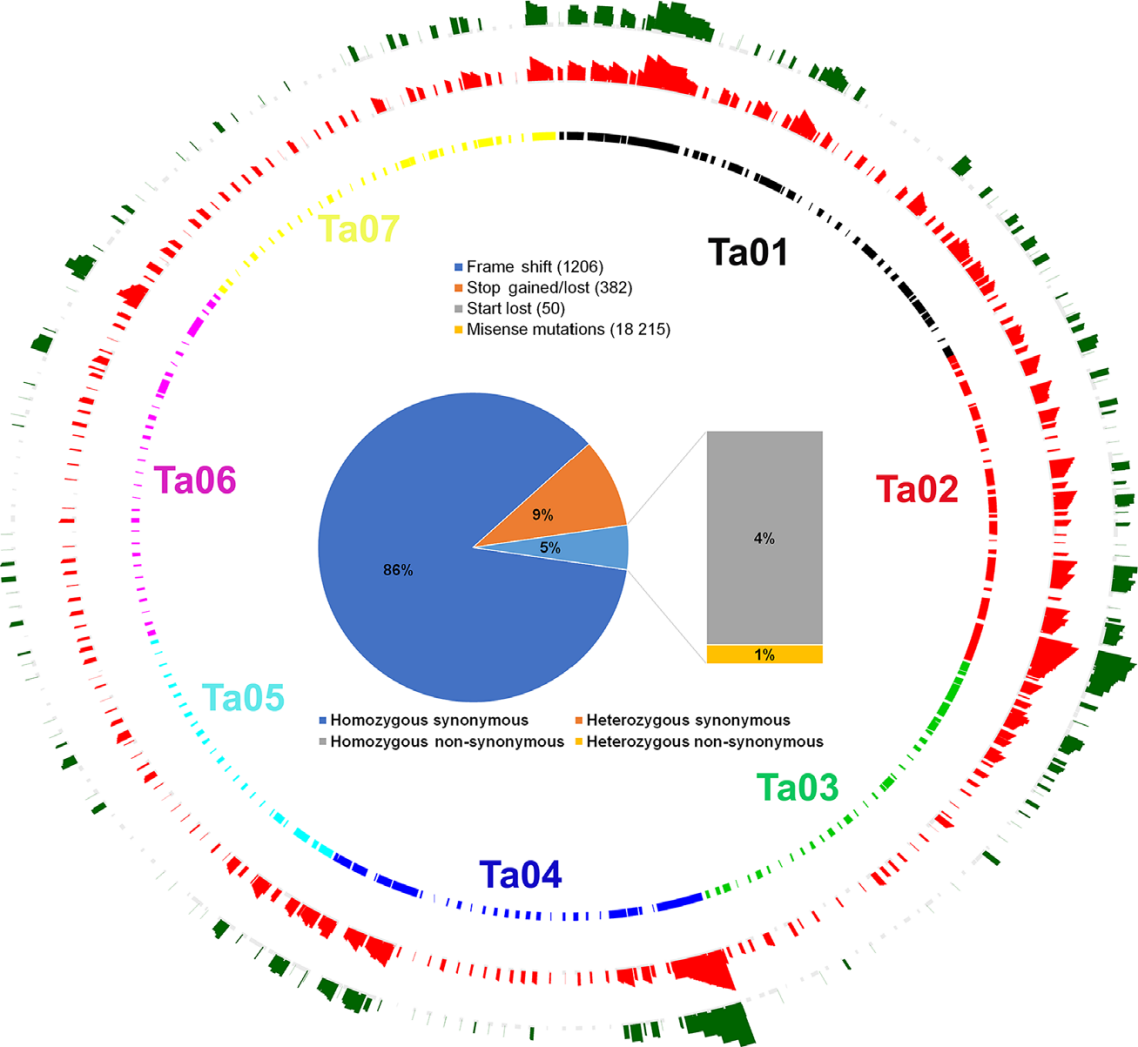
Circos plots representing distribution of 308 *Thlaspi arvense* reference genome scaffolds mapped to the Eutrema pseudo chromosomes, and the distributions of variants differentiating the Spring 32‐10 and MN106 genomes. Green represents non‐synonymous indels while red represents non‐synonymous single nucleotide variations between the Spring 32‐10 and MN106 genome sequences. The inner circle represents arrangements of pennycress scaffolds on the Eutrema pseudo chromosomes based on the synteny. The pie chart represents the percentage of variants classified as non‐synonymous and synonymous.

Mapping of the Spring 32‐10 genome sequences to the Eutrema pseudo‐chromosomes showed that the Spring 32‐10 versus MN106 SNPs were distributed throughout the pennycress genome (Figure 2). A total of 389 890 synonymous and 19 853 non‐synonymous variants were found in the Spring 32‐10 genome when compared to MN106 genome sequences. Non‐synonymous mutations were found in 6357 unique genes and constituted the classes of single amino acid substitution (18 215), frame shift (1206), translational stop gain/lost (382) and translational start lost (50) (Figure 2 and Table S2). We found that 3478 of 6357 genes had more than one variant, some of which could affect the gene lengths in the Spring 32‐10 genome.

We further compared the mutations from six other spring‐type lines sequenced by (Dorn *et al*., 2017) to identify variants specific to Spring 32‐10, identifying 5768 mutations present in 2082 unique genes (Table S3). Non‐synonymous variants unique to Spring 32‐10 were found in genes known to be involved in growth habit traits such as *DELAY OF GERMINATION1* (*DOG1)*; (Bentsink *et al*., 2006), *BRASSINOSTEROID INSENSITIVE1* (*BRI1*); (Friedrichsen *et al*., 2000), *PHYTOCHROME A* (*PHYA*); (Zhong *et al*., 1997), *PHYTOCHROME INTERACTING FACTOR4* (*PIF4*); (Kumar *et al*., 2012) and *TIME FOR COFFEE* (*TIC*); (Hall *et al*., 2003). Further studies must be done to establish functional roles.

The Spring 32‐10 sequences and metadata are available online in the NCBI Sequence Read Archive (SRA) under experiment SRX4066381 as run SRR7146892, at https://www.ncbi.nlm.nih.gov/Traces/study/?acc=SRP036068. The Spring 32‐10 versus MN106 genome variants were integrated into the *Thlaspi* version 1 genome browser and are viewable at http://pennycress.umn.edu/. We also sequenced genomic DNA from a Spring 32 plant that was not inbred (a plant from the originally collected Spring 32 population), using the Illumina HiSeq 2000 platform (100 bp, paired‐end reads). These non‐inbred Spring 32 sequences and metadata are available online in the NCBI SRA under experiment SRX877201 as run SRR1803284, at https://www.ncbi.nlm.nih.gov/sra?linkname=bioproject_sra_all&from_uid=275151.

### Pennycress transformation using an *Agrobacterium*‐mediated floral dip method requires vacuum infiltration

To determine if pennycress could be genetically transformed, we employed *Agrobacterium tumefaciens* strain GV3101 and various binary vectors harbouring different marker genes conferring *in planta* selection or visualisation. Those marker genes included hygromycin phosphotransferase II (*HPTII*) conferring resistance to hygromycin B, neomycin phosphotransferase II (*NPTII*) conferring resistance to kanamycin or its analogue paromomycin, Bialaphos resistance (bar) conferring resistance to glufosinate, and DsRed which produces a visible red fluorescent protein.

**Figure 3:**
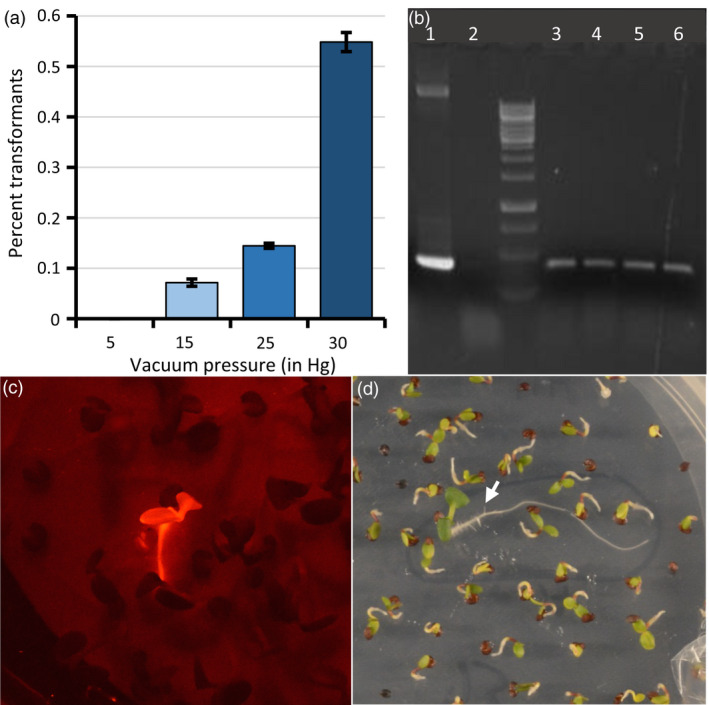
*Agrobacterium*‐mediated floral dip transformation of pennycress. (a) Transformation efficiencies associated with exposing pennycress racemes submerged in binary‐vector‐carrying Agrobacterium to various vacuum pressures for 5 min. (b) PCR analysis to confirm that red‐fluorescing seedlings were transgenic for the DsRed gene. Shown is agarose gel‐electrophoresced DsRed gene‐derived PCR products, using the following templates: Lane 1: DsRed gene‐containing binary vector DNA; 2: Tissue preparation from wild type; 3 through 6: Tissue preparations from four independent DsRed transformants. (c) A DsRed‐fluorescing transgenic pennycress seedling among non‐transgenic seedlings on an agar plate, detected using a NightSea fluorescent protein flashlight. (d) A transgenic pennycress seedling (arrow) carrying the *HPTII* gene and exhibiting resistance to 40 U/mL hygromycin B in an agar medium.

*Agrobacterium* cultures transformed with each of these binary vectors were suspended in a solution of 5% sucrose and 0.02% Silwet L‐77 (a surfactant), into which racemes of Spring 32 inflorescences that had been flowering for about 5 days (Figure S4) were submerged for duration ranging from 5 to 30 min under a range of vacuum pressures. Screening through thousands of the T_1_‐generation seeds arising from these plants, using the corresponding drug selection or DsRed fluorescence visualisation, revealed no drug resistant or fluorescent seedlings when no vacuum was applied, signifying that transformation had not occurred. However, for racemes that had been placed under vacuum while submerged in the *Agrobacterium* solution, transformants among the T_1_ seeds were identified, with the highest transformation efficiency (0.5%) corresponding with the highest vacuum pressure applied (30 in mercury (Hg), or 14.7 psi; Figure 3a). We observed no obvious differences in transformation efficiencies with vacuum durations longer than 5 min.

Of the various transformation selection protocols used, DsRed fluorescence screening using a Nightsea dual fluorescent protein flashlight allowed for relatively easy visual identification of transformants at the seedling or adult stages (Figures 3c and S5). Selection of seedlings transformed with the *HPTII* gene using 40 U/mL hygromycin B, also worked well, with transformants exhibiting sustained root and shoot growth and lack of cotyledon and leaf yellowing on drug‐containing agar media compared to untransformed germinating seedlings (Figures 3d and S6A). We found that wild‐type pennycress seedlings were naturally partially resistant to kanamycin as signified by prolonged and uneven root and shoot growth on agar media containing relatively high drug concentrations (Figure S6B), which made it difficult to distinguish transformed seedlings from those untransformed. However, pennycress seedlings exhibited more uniform sensitivity to the kanamycin analogue paromomycin at a concentration of 100 μg/mL (Figure S6C), allowing for more accurate identification of seedlings transformed with a T‐DNA vector containing the *NPTII* gene. While selection of *Bar* gene‐transformed seedlings grown on agar media containing 18 μg/mL glufosinate (119.7 μL/1000 mL Finale herbicide) allowed for relatively easy detection of transformants (Figure S6D), we caution against using this selectable marker being that glufosinate is a commonly used broad‐spectrum systemic herbicide, and there is a risk that glufosinate‐resistant pennycress seeds could inadvertently be released into the wild. It is important to note that, in the U.S., planting of transgenic pennycress seeds/plants outdoors or transportation across state lines is prohibited without USDA APHIS approval.

Along with Spring 32, we used the *Agrobacterium*‐mediated floral dip transformation method on four winter‐type pennycress cultivars to assess if winter‐type cultivars could also be transformed and if there were differences in transformation efficiencies. While all cultivars could be transformed, efficiencies varied from 0.06% to 0.39%, which were all less than the 0.55% efficiency of Spring 32 (nine transformants identified out of 2300 seeds from dipped plants for Elizabeth, five out of 1650 for a proprietary breeding line, one out of 1800 for W12, and one out of 1200 for Beecher, compared to 10 out of 1824 for Spring 32).

### CRISPR‐Cas9‐induced mutations in the putative *TaFAE1* gene abolish erucic acid content in pennycress seed triacylglycerols

Seeds of the various pennycress isolates we analysed contained from 24% to 39% oil by dry‐weight, in the form of triacylglycerols (TAGs) (Figures S1 and S2) (Moser *et al*., 2009a,b). TAGs function as energy stores for seeds in Brassicaceae species and are composed of three fatty acids of varying carbon lengths and degrees of saturation that are ester‐linked to the *sn*‐1, *sn*‐2 and *sn*‐3 positions of a glycerol backbone (Durrett *et al*., 2008). We found that 27%–39% of the fatty acids in the pennycress seed TAGs were of the very long chain monounsaturated fatty acid erucic acid (Figures S1 and S2). Erucic acid (C22:1) is synthesised from oleoyl‐CoA (C18:1‐CoA) via two successive elongation reactions carried out by the cytosolic fatty acid elongation complex (Millar and Kunst, 1997). It has been shown in other plant species that knockout mutations in the *FATTY ACID ELONGATION 1* (*FAE1*) gene, which encodes the ß‐ketoacyl‐CoA synthase responsible for the condensation reaction, result in the abolishment of seed oil erucic acid content (James and Dooner, 1990; James *et al*., 1995; Lemieux *et al*., 1990; Roscoe *et al*., 2001).

The *fae1* loss‐of‐function mutations are what give canola its ‘Zero Erucic’ trait, which makes the oil edible (Wu *et al*., 2008). Erucic acid is also an undesirable component in biodiesel owing to its high kinematic viscosity, which confers poor cold‐flow properties (Moser, 2012). Hence, it is a high priority to produce commercial pennycress varieties with the Zero Erucic trait. Since wild pennycress isolates contain seed oil erucic acid content much higher than can be reduced to food regulatory limits by breeding (Figures S1 and S2), we employed CRISPR‐Cas9 genome editing to knockout *FAE1* function.

To identify the putative *TaFAE1* gene orthologue in pennycress, we searched the *Thlaspi arvense* genome using a pennycress‐specific Basic Local Alignment Search Tool (BLAST) program we developed and made freely available at http://pennycress.umn.edu/. *Arabidopsis thaliana AtFAE1* gene sequences were used as the query. We also searched the *Thlaspi arvense* transcriptome shotgun assembly (TSA); (Dorn *et al*., 2015) using the National Center for Biotechnology Information (NCBI) nucleotide BLAST online search tool. Together, these searches identified the putative *TaFAE1* gene and the TSA sequence GAKE01018976.1 (https://www.ncbi.nlm.nih.gov/nuccore/GAKE01018976). The *TaFAE1* gene is predicted to have no introns and an open reading frame (ORF) 1521 nucleotides in length sharing 87.8% nucleotide sequence identity with the 1521 bp *AtFAE1* ORF (AT4G34520) (Figure S7).

We created a CRISPR‐Cas9 binary vector construct named *TaFAE1*‐CRISPR‐Cas9_Hyg, utilising a vector set optimised for use in Arabidopsis (Fauser *et al*., 2014). The *TaFAE1*‐CRISPR‐Cas9_Hyg vector was designed to target edits ~215 bp downstream of the *TaFAE1* translational start site (Figure S7).

**Figure 4:**
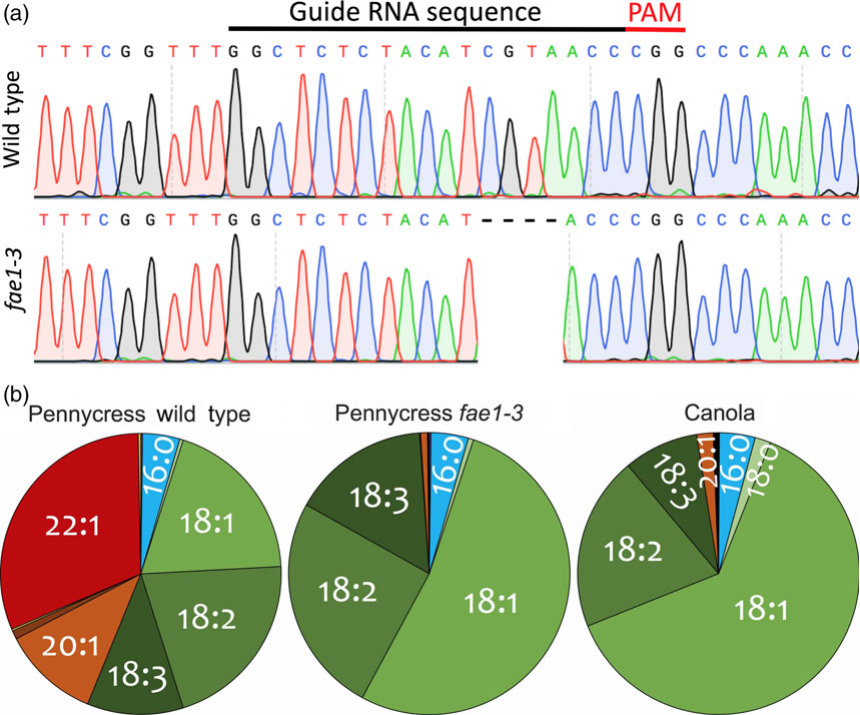
The pennycress *fae1‐3* mutant harbours a 4‐bp deletion in the *TaFAE1* coding sequence, producing seed oil (triacylglycerols; TAG) with undetectable amounts of erucic acid (C22:1) and greatly reduced amounts of eicosenoic acid (C20:1). (a) DNA sequence chromatograms showing the location of the *fae1‐3* 4‐bp deletion in relation to the CRISPR‐SpCas9 protospacer and adjacent PAM site. (b) Pie charts showing relative amounts of fatty acids in pennycress wild type, pennycress *fae1‐3* mutant and canola seed TAGs.

The *TaFAE1*‐CRISPR‐Cas9_Hyg vector was introduced into pennycress plants using the *Agrobacterium*‐mediated vacuum infiltration method described above. To identify plants with Cas9 editing activity, we performed T7 endonuclease I screening (Pyott *et al*., 2016) of *FAE1* PCR products amplified from leaf genomic DNA. T7 endonuclease I cuts double‐stranded DNA at sites where there is DNA base‐pair mismatching, in this case originating from CRISPR‐Cas9‐induced mutations. We generated 11 independent T_1_‐generation plants using this vector construct and performed T7 endonuclease I analysis on at least 14 T_2_ progeny from three of those T_1_ plants. This analysis showed that only progeny from one of the three T_1_ plants had Cas9 editing activity at the *FAE1* gene target site; that activity was confirmed by DNA sequence analysis of *FAE1* PCR products. The majority of the *fae1* mutations in these plants were indecipherable, probably due to cells within the leaf tissue having different indel mutations leading to ‘gobbledygook’ sequence starting at the CRISPR target site. Most of those mutations were not inherited in the next generation, suggesting the mutations likely originated in vegetative cells and not in germline cells. We focused on one T_2_ plant clearly heterozygous for a 4 bp *fae1* deletion. T_3_ progeny of that plant segregated in a Mendelian fashion for the 4 bp deletion while at the same time exhibited new *fae1* mutations likely arising in the other *FAE1* allele. By sequencing 26 T_4_ progeny arising from those T_3_ plants, we identified a plant homozygous for a single A insertion and a plant homozygous for a single T deletion in *FAE1* along with plants homozygous for the 4 bp deletion (Figures 4a and S8A). Since these three mutations were stable and inherited in subsequent generations, we named the corresponding *fae1* homozygous lines *fae1‐3* (4 bp deletion), *fae1‐4* (single A insertion) and *fae1‐5* (single T insertion). In retrospect, to minimise the cost and time of identifying Cas9‐active lines, we recommend sequencing CRISPR target‐site sequences in all T_1_‐generation plants.

Seeds from homozygous *fae1‐3*,* fae1‐4* and *fae1‐5* plants were analysed for TAG fatty acid composition, revealing negligible amounts of erucic (C22:1) and eicosenoic (C20:1) acids in all three lines along with elevated amounts of oleic (C18:1), linoleic (C18:2) and linolenic (C18:3) acids (Figures 4b and S8B). This fatty acid profile is comparable to that of canola oil albeit with higher amounts of the polyunsaturated linoleic and linolenic acids (Figure 4b).

### Pennycress seeds expressing *EaDAcT* accumulate acetyl‐TAG

The ease by which pennycress could be genetically transformed suggested pennycress could be used as a malleable platform for production of unusual lipids. To demonstrate this capability, and to further demonstrate the efficacy of the developed pennycress transformation method, we transformed the gene encoding the *Euonymus alatus* diacylglycerol acetyltransferase (*Ea*DAcT) under the control of the soybean glycinin promoter, into wild‐type pennycress plants. *Ea*DAcT incorporates an acetate moiety in the *sn*‐3 position of TAG resulting in an oil with reduced‐viscosity properties (Durrett *et al*., 2010).

**Figure 5:**
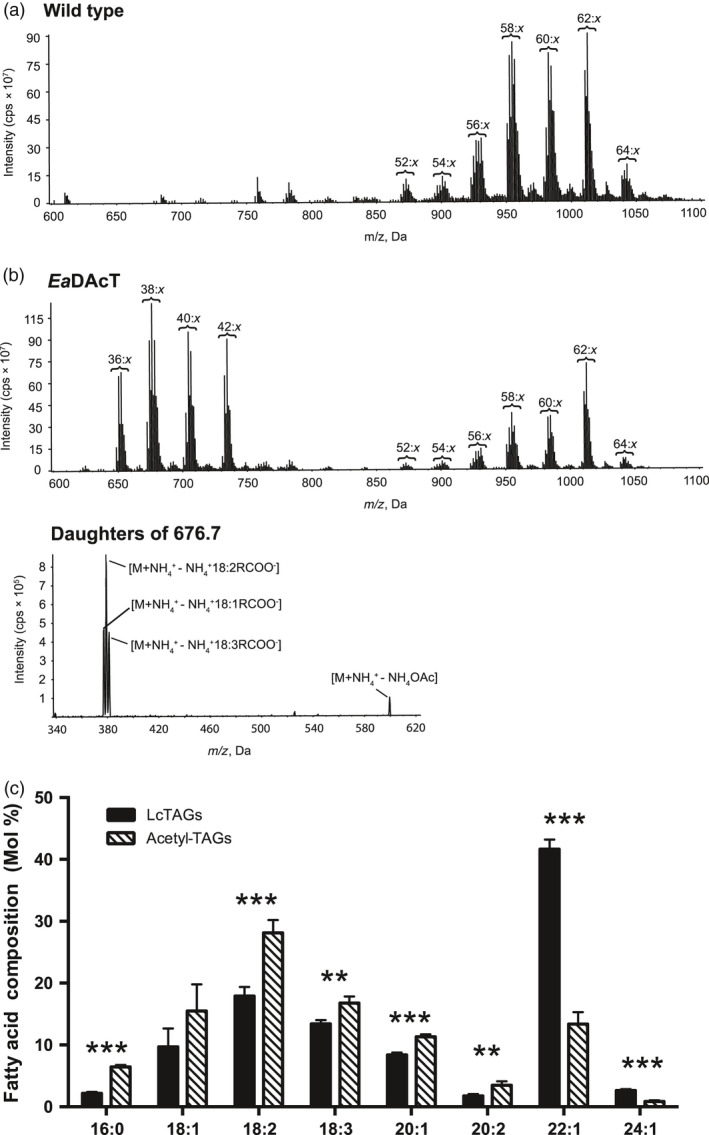
Pennycress seeds expressing *Ea*DacT accumulate acetyl‐TAGs. (a) Positive ESI mass spectra of neutral lipids from pennycress wild‐type seed or transgenic T_3_ seed expressing *Ea*DAcT. Signal peaks possess the m/z value of [M+NH_4_]^+^ adduct. For clarity, only the number of acyl carbons and not the number of double bonds (x) in each series of acetyl‐TAG and lcTAG molecular species is indicated. (b) ESI‐MS^2^ daughter scans of acetyl‐TAGs from Pennycress seed expressing *Ea*DAcT. Shown are the fragment peaks for acetyl‐TAGs with [M+NH_4_]^+^ adducts with mass of 676.7. (c) Mean fatty acid composition of acetyl‐TAGs and endogenous lcTAGs present in the T_3_ seed of four independent transgenic lines expressing *Ea*DAcT. Error bars represent SEM. Asterisks indicate statistical significant (Student's *t*‐test, **, *P* < 0.01; ***, *P* < 0.01).

Similar to previous work in other plant species (Durrett *et al*., 2010; Liu *et al*., 2015), transformation of *Ea*DAcT resulted in the accumulation of acetyl‐TAGs in pennycress seeds as determined by electrospray ionisation mass spectrometry (ESI‐MS; Figure 5a). Product ion analysis generated daughter fragments consistent with the loss of an acetate group (Figure 5b), confirming the identity of the acetyl‐TAGs in the transformed lines. Compared to the regular TAGs still produced in the transgenic seeds, the acetyl‐TAGs contained less than a third of the amount of C22:1, but increased levels of most other fatty acids (Figure 5c). Consistent with this observation, acetyl‐TAGs synthesised in other transgenic Brassicaceae also contain lower levels of very long chain fatty acids (VLCFA) (Durrett *et al*., 2010; Liu *et al*., 2015). This much lower incorporation of C22:1 in acetyl‐TAGs is likely explained by an increased preference of this VLCFA for the *sn*‐3 position of TAG, similar to the situation in other Brassicaceae species (Takagi and Ando, 1991; Taylor *et al*., 1995). As the *sn*‐3 position in acetyl‐TAGs is occupied by acetate, it is unavailable for acylation by VLCFA such as 22:1 that would typically be incorporated there, leading to lower levels of this fatty acid in acetyl‐TAGs.

## Discussion

The adoption of *Arabidopsis thaliana* as a model system was transformational to plant science, allowing for rapid discoveries related to many aspects of plant growth, development and abiotic and biotic stress responses at the organismal and cellular levels (Flavell, 2009; Provart *et al*., 2016; Somerville and Koornneef, 2002). As impactful as Arabidopsis has been, its diminutive stature and lack of agronomic potential has limited its utility as a model to address questions pertaining to crop performance. Here, we introduce *Thlaspi arvense* (pennycress) as a new model system that is as easy to grow and manipulate as Arabidopsis, and which itself can serve as a major oilseed crop with ecosystem services benefits. Experiments can be performed on pennycress that directly test and/or improve agronomic performance, allowing for combining basic with applied research.

Pennycress's relatively small diploid genome along with a mostly one‐to‐one gene correspondence with Arabidopsis allows for relatively easy and rapid functional analyses. Given the relative ease of growing pennycress as single plants or in plots outdoors (Figure 1), pennycress natural variants and mutants can serve as powerful tools in dissecting environmental responses as well as interactions with other organisms including the microbiome. In the laboratory, the larger cell and organ sizes of pennycress compared to Arabidopsis (Figures 1 and S9) will help facilitate a variety of related research, from live‐cell imaging to biochemical analyses to omics.

Although pennycress naturally grows in farm fields, e.g. throughout the U.S. Midwest region where it is being tailored to serve as a cover crop, pennycress is not considered a problem weed by Midwest farmers due to its off‐season lifecycle and ease of control by tilling or commonly used herbicides. Field trials in Illinois and Minnesota have demonstrated that pennycress has a minimal impact on subsequently planted soybeans (Johnson *et al*., 2015; Phippen and Phippen, 2012). Moreover, pennycress is not invasive to established ecosystems, but instead grows where the ground has been disturbed.

We produced an inbred line of pennycress named Spring 32‐10, the progenitor seeds of which originated from a natural population of spring annual plants collected near Bozeman, Montana. Spring 32‐10 is the culmination of ten rounds of self‐pollination and single seed descent. Selections were made each generation for seeds and plants that germinated and grew well under laboratory conditions. By definition, spring‐type plants do not require cold treatment to flower (vernalisation), which allows for more rapid experimental progress than is possible with winter‐type pennycress isolates such as MN106, Beecher and Elizabeth, which require e.g. ~4 °C treatment for ~21 days in the laboratory or over‐wintering of plants in the field to induce flowering.

Spring 32 isolates including Spring 32‐10 perform consistently well both in laboratory and field settings. Seed germination and plant growth requirements are comparable to those of Arabidopsis. For example, Spring 32 seeds have low seed dormancy and can be planted immediately after seed harvest without GA treatment, allowing for rapid generation‐to‐generation experimentation. Plant generation time can be accelerated by limiting or not applying nitrogen fertiliser, allowing for seed‐to‐seed growth in as little as 9 weeks (Table 1 and Figure S3). Generation time may also be reduced e.g. by growing the plants in smaller pots or under crowded conditions and/or by using longer day‐lengths (e.g. 22 h) and relatively higher light intensities (e.g. mixed fluorescent/incandescent lighting at ~300 μE).

To facilitate translational research, we sequenced the genome of the Spring 32‐10 line as well as that of a Spring 32 non‐inbred plant (see Results and Materials and Methods sections for additional details). The Spring 32‐10 sequences are publicly available in the NCBI Sequence Read Archive (SRA) as accession SRP036068; the sequences of other pennycress spring‐type lines characterised by Dorn *et al*. (2017) can be found there. The Spring 32 non‐inbred genome sequences are available in the NCBI SRA as accession SRR1803284.

The Spring 32‐10 genomic sequences were compared to those of line MN106 to identify natural variants. MN106 genome sequences serve as the reference genome (*Thlaspi* version 1; (Dorn *et al*., 2015) and are publicly available/searchable at http://pennycress.umn.edu/, while MN106 transcriptome sequences are publicly available/searchable through the NCBI Transcriptome Shotgun Assembly (TSA) (Dorn *et al*., 2013). In addition, the variations between Spring 32‐10 and MN106 sequences identified by this study can be found in Table S2 and have been incorporated into the pennycress genome browser at http://pennycress.umn.edu/. Spring 32‐10 inbred‐line seeds are available from the Arabidopsis Biological Resource Center (ABRC).

Dorn *et al*. (2017) recently explored the basis of the pennycress spring‐type flowering habit by performing whole‐genome sequence analysis on accessions collected throughout North America and Europe. Interestingly, it was found that in all cases the loss of vernalisation requirement was due to mutations in the *FLOWERING LOCUS C* (*FLC*) gene. Four different alleles (*flc‐A* through *flc‐D*) were identified thereby showing the spring‐type flowering habit arose multiple times in natural populations. We found the Spring 32‐10 genome harbours the *flc‐B* mutant allele, which is a 456 bp deletion that removed the entire second exon of the *FLC* gene.

Comparisons of Spring 32‐10 whole‐genome sequences to winter annual MN106 draft genome sequences (Dorn *et al*., 2015) identified 409 743 variants comprised of 360 186 SNPs and 49 557 INDELs. Of those, 1637 heterozygous and 18 216 homozygous non‐synonymous variants residing in 6357 unique genes were identified, which by their nature could be the source for natural variation observed in the phenotypes. We identified 5768 non‐synonymous variants residing in 2082 unique genes that were specific to Spring 32‐10, after removing the known variants in the other spring‐type genome sequences from the Dorn *et al*. (2017) study (Table S3). Some of these variants are in important genes or gene families which might help researchers better understand spring and winter annual growth habits as well as how traits evolved in different habitats. For example, genes in addition to *FLC* controlling traits that differentiate winter and spring annuals include those related to flowering time (Dorn *et al*., 2017) as well as sensitivity to temperature and light. Notably, we identified Spring 32‐10 non‐synonymous mutations in clock‐controlled and light dependent genes such as *TIC* (Hall *et al*., 2003) and *PHYA* (Zhong *et al*., 1997). In addition, some of the Spring 32‐10 genes predicted to be important for domestication such as *DOG1* (germination) (Kendall *et al*., 2011) and *MYB118* (glucosinolate content) (Zhang *et al*., 2015) also had a mutation in each. Further studies are needed to establish the functional roles of this natural variation. Based on studies of related genes in Arabidopsis and other Brassicaceae, it is highly likely that at least some of these identified mutations alter gene function and confer phenotypes.

We show that pennycress can be genetically transformed by employing a relatively simple *Agrobacterium* floral dip method that generates as much as 0.5% transformed seeds. Unlike Arabidopsis, but like camelina, application of a vacuum during the floral dip process is required for transformation to occur (Clough and Bent, 1998; Lu and Kang, 2008). We also demonstrate that pennycress is amenable to CRISPR‐Cas9 genome editing. CRISPR‐Cas9 is a relatively new and simple genome editing tool that introduces deletions and/or insertions at a chromosomal location chosen by the researcher (Belhaj *et al*., 2013).

For this study, we employed CRISPR‐Cas9 DNA vectors originally designed for use in Arabidopsis (Fauser *et al*., 2014), containing the *Streptococcus pyogenes Cas9* (*SpCas9*) endonuclease gene. We targeted CRISPR‐SpCas9‐induced mutations in the *TaFAE1* gene to abolish erucic acid biosynthesis in pennycress seed oil thereby making an edible oil. This so‐called ‘zero erucic’ trait is a defining feature of canola oil; *fae1* loss‐of‐function mutations are also the basis of this trait in canola (Wu *et al*., 2008). While erucic acid has industrial value in the manufacture of polyethylene sheets, as a heat‐stabilising component in lubricants, and as a feedstock for jet fuel (Nieschlag and Wolff, 1971), it is not desirable for human and animal consumption given its association with myocardial lesions and abnormal fat accumulation in rat feeding studies (Hung *et al*., 1977). Moreover, erucic acid is undesirable in diesel fuels due to its poor cold‐flow properties (Knothe, 2005; Moser *et al*., 2009a,b). Therefore, this genetic change in pennycress is essential for attaining oil suitable for food, feed and biodiesel uses.

The ability to easily transform pennycress enables the use of synthetic biology approaches to further alter the oil profiles of the seeds, enabling the production of the many unusual fatty acids and storage lipid structures found throughout the plant kingdom. Many of these unusual lipids possess chemical and physical properties useful for different applications. For example, the presence of the *sn*‐3 acetate group in the acetyl‐TAGs synthesised by the seeds of *Euonymus alatus* (Burning bush) means that these unusual storage lipids have a lower kinematic viscosity and improved cold temperature properties compared to regular vegetable oils, enabling applications such as improved drop‐in biofuels or biolubricants (Durrett *et al*., 2010; Liu *et al*., 2015). Acetyl‐TAGs are synthesised by a diacylglycerol acetyltransferase named *Ea*DAcT that transfers a two‐carbon acetate group to diacylglycerol instead of a long acyl chain. Similar to what has been demonstrated in other plant species, expression of *Ea*DAcT in pennycress results in the accumulation of acetyl‐TAGs in the seed of transgenic lines (Figure 5).

The ability to easily engineer pennycress to produce these useful lipids and then grow high yielding lines in the field will allow the production of large quantities for functional testing. This approach can obviously be extended to enable pennycress to commercially produce a variety of industrially useful lipids through the expression of the appropriate lipid biosynthetic genes. For example, combining the synthesis of medium chain fatty acids with that of acetyl‐TAGs should result in novel molecules predicted to possess further reductions in their kinematic viscosity, allowing for direct drop‐in use in the diesel engines of commercial cars, trucks, tractors and tractor‐trailers (Aznar‐Moreno and Durrett, 2017).

In addition to the resources and tools highlighted here, large pennycress EMS mutant populations have been generated, and screens have so far identified Arabidopsis‐like mutants and corresponding mutations in orthologous genes controlling many traits, supporting the hypothesis of one‐to‐one gene correspondence (Marks *et al*., submitted). Given the amenability of pennycress to genetic improvements and experimentation, it is not unreasonable to conclude that pennycress can be domesticated as a new crop with unprecedented speed, and will serve as a useful model, including for translational research leading to agronomic improvements to its less user‐friendly polyploid oilseed relatives, rapeseed canola, camelina and carinata.

## Materials and methods

### Pennycress variety information

The pennycress ‘Spring 32’ population of seeds was collected near Bozeman, Montana (Lat: 45.664, Long: −111.048, Alt: 1496 (m)). Seeds of the winter‐type cultivar ‘Elizabeth’ were obtained from Dr. Terry Isbell at the USDA‐ARS (Peoria, IL), who selected Elizabeth from the wild population ‘Beecher’ as having enhanced germination in both light and dark conditions (Isbell *et al*., 2017). Beecher seeds, which were collected by Dr. Terry Isbell near Hanna City, IL (Lat: 40.7150, Long: −89.796, Alt: 177 (m)), as well as Elizabeth seeds are available as accessions PI 672505 and PI 677360 respectively, from the USDA‐ARS Germplasm Resources Information Network (GRIN), (https://npgsweb.ars-grin.gov/gringlobal/search.aspx, search term pennycress). Elizabeth, Beecher and most other pennycress winter‐types require vernalisation at 4 °C for ~21 days, as seedlings or as plants, to induce flowering.

### Surface sterilisation of pennycress seeds and growth conditions

Pennycress seeds were surface sterilised with a brief rinse of 70% ethanol followed by a 10‐minute incubation in a sterilisation solution consisting of 30% bleach and 0.01% SDS. After sterilisation solution removal, the seeds were rinsed three times with sterile water. For pennycress varieties having primary seed dormancy (e.g. Beecher), to break dormancy, after the third water rinse and before plating, seeds were left to soak at least 30 min in 0.01 mm gibberellin 4+7. (GA_4+7_, PhytoTechnology Laboratories product no. G358; GA_4+7_ powder was initially dissolved in 95% ethanol then diluted in water to make a 1 mm stock solution; the 10 μm working solution (made from the stock solution) was used within 2 weeks of being made). Gibberellin treatment was not necessary for Spring 32 seed germination as it has low primary seed dormancy.

Surface‐sterilised pennycress seeds were sown onto 0.8% agar media containing one‐half‐strength Murashige and Skoog salts, or onto moistened Whatman 3MM chromatography paper (cat# 3030‐6461), in Parafilm‐wrapped Petri dishes, then immediately placed into a Percival Scientific CU‐36L5 incubator (16 h 4100K fluorescent light ~150–200 μE/m^2^/s/8 h dark, 22^o^ C). For growth in soil, seedlings were transplanted at a density of four plants per 4‐inch pot (Gage Dura Pots 4″ x 3‐3/8″, OBC Northwest Inc. catalog no. PPG4) in autoclaved Redi‐Earth plug and seedling soil mix (or a 50/50 mix of Redi‐Earth plug and seedling mix and Berger BM 7 bark mix) intermixed with 0.03 g/4‐inch pot of the insecticide Marathon (www.domyownpestcontrol.com/ OHP Marathon 1% Granular). When making up the 4‐inch pots, a thin layer of wet soil (~1/4 inch) was first put in the bottom of the pot, on top of which 1/8 teaspoon of prilled urea (46‐0‐0; Greenway Biotech, Inc.) was sprinkled before the pot was entirely filled with the wet soil mix. Plants were grown in environment‐controlled growth chambers cycling 16 h light/8 h dark (light was either 6500K fluorescent or a combination 4100K fluorescent/incandescent lighting, 175–250 μE/m^2^/s light intensity), at 21 or 22 °C.

### Spring 32‐10 whole‐genome sequencing

Whole‐genome sequencing was performed on DNA isolated and pooled from six 10th‐generation inbred seedlings using Illumina HiSeq 2500 at the University of Minnesota Genomics Center. Paired‐end raw reads were processed through Trimmomatic (Bolger *et al*., 2014) to remove the adaptors and low‐quality reads, then mapped to and aligned with the assembled *Thlaspi* v1.0 MN106 genome sequences (Dorn *et al*., 2015) using BWA tools (Li and Durbin, 2009). Aligned files were passed through SAMtools (Li *et al*., 2009) and Picard tools (http://broadinstitute.github.io/picard/) to remove PCR duplicates and assign read groups to the files. Genomic variations in the Spring 32‐10 versus MN106 genome sequences were identified using HaplotypeCaller in the Broad Institute's Genome Analysis Toolkit (McKenna *et al*., 2010), which is effective in identifying the INDELs along with the Single Nucleotide Polymorphisms (SNPs). To obtain high confidence variants, we excluded SNPs or INDELs with the read depth (DP) of ≤20 and Quality (QD) of ≤20. Selected variants were then compared to the *Thlaspi* v1 coding sequences (CDS) and protein database to identify the variants in the coding regions. To examine the distribution of variants in the Spring 32‐10 genome, a synteny between the *Eutrema salsugineum* (Eutrema) and *Thlaspi arvense* (pennycress) genomes was constructed using a Syntenic Path Assembly in SynMap (DAGChainer—Relative Gene order, −D = 20, −A = 5, skip random/unknown chromosomes).

Synonymous and non‐synonymous SNPs and INDELs (Spring 32‐10 versus MN106) were mapped using Circos Plot (Hu *et al*., 2014) from R (version 3.2). These polymorphisms are listed in Table S2 and can also be publicly accessed in the genome browser at http://pennycress.umn.edu, which allows for highlighting all the variants identified in this analysis. To identify non‐synonymous mutations unique to Spring 32‐10, we used sequence information from other spring lines PI650287, PI650284, PI63341, PI633415, MN108 and Ames22461 (Dorn *et al*., 2017) to filter out natural variants occurring in wild populations.

### Vectors

The pGly*Ea*DAcT binary vector construct (previously described in (Durrett *et al*., 2010) possesses the *Euonymus alatus* diacyglycerol acetyltransferase (*EaDAcT*) gene driven by the seed‐specific *Glycine max* Glycinin promoter. The construct also contains the *NPTII* gene for 50 μg/mL kanamycin selection in bacteria and the *DsRed* gene driven by a Cassava Vein Mosaic Virus (*CVMV*) promoter for red florescent protein visual screening in plants (Nguyen *et al*., 2013). The *NPTII* gene‐containing pHAN binary vector (also called pHAN1) (Li *et al*., 2012) was used to transform pennycress to test kanamycin and paromomycin selection. For testing glufosinate selection, pennycress plants were transformed with a proprietary binary vector containing the *Bar* gene; the *Bar* gene sequences were identical to those in the pFGC‐pcoCas9 vector (Li *et al*., 2014).

The *TaFAE1*‐CRISPR‐Cas9_Hyg binary vector construct was generated, as described in (Fauser *et al*., 2014) and at http://www.botanik.kit.edu/molbio/940.php, using the vectors pEn‐Chimera and pDe‐Cas9. The plant selectable marker (*Bar* gene) in the pDe‐Cas9 binary vector was replaced with the *Hygromycin phosphotransferase* (*hpt*) gene (40 U/mL hygromycin selection in plants) to create a pDe‐Cas9_Hyg vector. Bacterial selection used for the binary vector was 75 μg/mL spectinomycin.

The following two oligos were annealed to create the 20‐mer protospacer specific to the open reading frame of the putative pennycress *TaFAE1* gene:

Penny*FAE1*_CRISPR_FWD: ATTGTGGCTCTCTACATCGTAACC

Penny*FAE1*_CRISPR_REV: AAACGGTTACGATGTAGAGAGCCA

Constructs were introduced into *Agrobacterium tumefaciens* strain GV3101 using a standard CaCl_2_ flash‐freeze/thaw transformation method (Holsters *et al*., 1978).

### Agrobacterium‐mediated transformation of pennycress

Cultures of *Agrobacterium tumefaciens* strain GV3101 containing either the pGly*Ea*DAct plasmid or *TaFAE*1‐CRISPR‐Cas9_Hyg plasmid were started from glycerol stocks (~200 uL inoculated into 50 mL Luria Broth (LB) containing 50 μg/mL gentamycin, 50 μg/mL rifampicin, plus either 50 μg/mL kanamycin for pGly*Ea*DAct or 75 μg/mL spectinomycin for *TaFAE*1‐CRISPR‐Cas9_Hyg). The 50 mL cultures were shaken overnight at 28 °C, then added to an additional 200 mL LB antibiotic‐containing media and again incubated overnight, then centrifuged at 3500 ***g*** for 10 min and resuspended in an equal volume of 5% (w/v) sucrose plus 0.02% (v/v) Silwet L‐77. The floral portion of inflorescences of plants that had flowers opening for ~5 days (see Figure S4A as an example) were submerged in this *Agrobacterium* solution, then placed under ~30 inches mercury (14.7 psi) vacuum in a 26 cm × 25 cm × 36.5 cm vacuum chamber for 5–10 min or as otherwise noted, using a diaphragm vacuum pump (60 L/min pump speed, 200 mBar ultimate vacuum) (Figure S4B,C). After dipping, the floral portions of the inflorescences were wrapped in plastic wrap sealed around the stems with twist ties, and the plants placed back into an environmental growth chamber. The plastic wrap covering was removed the following day (Figure S4D,E).

### Identification of transgenic and CRISPR‐Cas9‐edited pennycress plants

#### DsRed screening

Putative pGly*Ea*DAcT transgenic pennycress seeds were surface sterilised and germinated on either 0.8% agar media containing one‐half‐strength Murishige and Skoog salts or moist chromatography paper. After ~6 days of growth, seedlings were screened for DsRed expression using a NightSea Dual Fluorescent Protein Flashlight (DFP‐GC; https://www.nightsea.com/products/dfp/).

#### Antibiotic selection

Putative *TaFAE*1‐CRISPR‐Cas9_Hyg transgenic seeds were surface sterilised and plated onto 0.8% agar/one‐half‐strength Murashige and Skoog salts containing 40 U/mL hygromycin B. Seedlings that continued to grow on the antibiotic‐containing media (Figure 3d) were transferred to soil ~8 days after plating, then after establishment were confirmed as being transgenic by PCR analysis (Figure 3b).

#### Screening for CRISPR‐Cas9 edits

Leaf‐extracted genomic DNA was PCR amplified using *FAE1* primers spanning the CRISPR‐Cas9 target site (see Supporting Information), followed by T7 endonuclease I digestion of the PCR product as described by (Pyott *et al*., 2016). Template for PCR reactions was a 50:50 mix of each putative mutant prep and wild type to ensure that even in the case of a homozygous mutation, a DNA mismatch would be PCR amplified and detected. PCR template was extracted from fresh leaf tissue using the Phire Plant Direct PCR Kit (Thermo #F130WH). For T7 endonuclease I analysis, 10 μL of each 20 μL PCR reaction was denatured by heating at 95 °C for 5 min in a thermocycler (Fisher) and annealed using gradual cooling: −2 °C per second decrease from 95 to 85 °C then −0.1 °C per second decrease from 85 to 25 °C, followed by T7 endonuclease I (Fisher Scientific cat. #50‐995‐224 to New England Biolabs cat. #M0302L, Ipswich, MA) digestion for 30 min. The digested product was electrophoresed in a 1% agarose gel to identify samples that partially digested, indicating an SpCas9‐induced edit in *TaFAE1*, which were confirmed by Sanger sequence analyses.

### Lipid analysis

Total lipids were extracted from pennycress seeds, and fatty acid methyl esters (FAMEs) were generated and analysed by gas chromatography, as described in (Cahoon *et al*., 2006). For ESI‐MS analysis, neutral lipids were purified from total seed lipids on a small silica column with 99:1 (v/v) chloroform: methanol. Samples were then analysed on an API4000 triple quadrupole mass spectrometer (Applied Biosystems) as described previously (Bansal and Durrett, 2016). To quantify fatty acid composition, total seed lipids were separated using thin layer chromatography on Silica gel 60 plates (Merck) with a 70:30:1 hexane: diethyl‐ether: acetic acid (v/v/v) solvent system. Lipids were visualised by spraying with 0.075% 2′,7′‐dichlorofluorescein in 95% methanol and exposing to UV light. The bands were scraped and directly transmethylated using a base‐catalysed method (Ichihara *et al*., 1996); the resulting FAMEs were analysed using gas chromatography.

## Conflict of interest

Illinois State University (John Sedbrook) and the University of Minnesota (M. David Marks) have entered licensing agreements with Arvegenix, Inc. for use of the pennycress *fae1* germplasm. John Sedbrook has stock in Arvegenix, Inc.

## Authors contribution

MM, BJ and ME developed and optimised the pennycress floral dip transformation and selection methods. WP and MP isolated pennycress wild populations and performed related phenotypic analyses. RC and KD performed sequence analyses on Spring 32‐10 and MN106 genomes. MM and BJ generated and characterised the *fae1* CRISPR‐Cas9 mutants. SB and TD generated the *EaDAcT* constructs and performed the Acetyl‐TAGs analyses. TN performed the fatty acids analyses. MM, WP, RC, EC, TD, MDM and JS designed and interpreted experiments, and wrote and edited the manuscript.

## Supporting information


**Figure S1** Collection locations and seed characteristics for wild pennycress populations.
**Figure S2** Seed oil and weight characteristics of 34 USDA accessions.
**Figure S3** Development of pennycress in soil amended with five nitrogen amounts.
**Figure S4** *Agrobacterium*‐mediated floral transformation of pennycress.
**Figure S5** Visualisation of red fluorescence from DsRed transgenic pennycress plants.
**Figure S6** Dose responses of pennycress seedlings grown on selection media.
**Figure S7** Nucleotide sequence alignment of the *AtFAE1* and *TaFAE1* ORFs.
**Figure S8** DNA sequence chromatograms of the CRISPR‐Cas9 induced *fae1* mutations.
**Figure S9** Microscopic images comparing Arabidopsis versus pennycress root cell sizes.
**Appendix S1** Supporting Materials and Methods
Growth conditions for nitrogen dosage experiment.
Growing pennycress in the field.
PCR analyses including analysis of transgenic plants.

**Table S1** Annotation of 27 390 predicted genes in the pennycress Spring 32‐10 genome.

**Table S2** Polymorphisms between the MN106 reference genome and the Spring 32‐10 genome.

**Table S3** Polymorphisms unique to the Spring 32‐10 genome compared to six other pennycress spring‐type genomes.
