# Supplementary material for: Molecular tools enabling pennycress (Thlaspi arvense) as a model plant and oilseed cash cover crop
Source: Plant Biotechnol J. 2018 Oct 25;17(4):776–88. doi: 10.1111/pbi.13014 (PMC6419581; doi:10.1111/pbi.13014)
Supplement: Supplementary file 1 — Figure S1 Collection locations and seed characteristics for wild pennycress populations. Figure S2 Seed oil and weight characteristics of 34 USDA accessions. Figure S3 Development of pennycress in soil amended with five nitrogen amounts. Figure S4 Agrobacterium‐mediated floral transformation of pennycress. Figure S5 Visualisation of red fluorescence from DsRed transgenic pennycress plants. Figure S6 Dose responses of pennycress seedlings grown on selection media. Figure S7 Nucleotide sequence alignment of the AtFAE1 and TaFAE1 ORFs. Figure S8 DNA sequence chromatograms of the CRISPR‐Cas9 induced fae1 mutations. Figure S9 Microscopic images comparing Arabidopsis versus pennycress root cell sizes. Appendix S1 Supporting Materials and Methods Growth conditions for nitrogen dosage experiment. Growing pennycress in the field. PCR analyses including analysis of transgenic plants. [file PBI-17-776-s003.pdf]

Supporting Information

Molecular tools enabling pennycress (*Thlaspi arvense*) as a model plant and oilseed cash cover crop

Michaela McGinn,[1] Winthrop B. Phippen,[2] Ratan Chopra,[3] Sunil Bansal,[4] Brice A. Jarvis,[1] Mary E. Phippen,[2] Kevin M. Dorn,[3] Maliheh Esfahanian,[1] Tara J. Nazarenus,[5] Edgar B. Cahoon,[5] Timothy P. Durrett,[4] M. David Marks,[3] and John C. Sedbrook[1*]

[1] School of Biological Sciences, Illinois State University, Normal, IL 61790, USA

[2] School of Agriculture, Western Illinois University, Macomb, IL 61455, USA

[3] Department of Plant Biology, University of Minnesota, Saint Paul, MN 55108, USA

[4] Department of Biochemistry and Molecular Biophysics, Kansas State University, Manhattan, KS 66506, USA

[5] Center for Plant Science Innovation and Department of Biochemistry, University of Nebraska-Lincoln, Lincoln, NE 68588, USA

[*]To whom correspondence should be addressed:

John Sedbrook
School of Biological Sciences
Campus Box 4120
Illinois State University
Normal, IL 61790 USA
phone 309-438-3374
fax 309-438-3722
email: jcsedbr@ilstu.edu

Supporting Materials and Methods

Growth conditions for nitrogen dosage experiment

Growth chamber conditions were set to an 18-hour photoperiod (7.9µE/m2/sec) with day/night temperatures of 24°C and 20°C, respectively. Seeds were sown directly onto the surface of wet soil (Pro-Mix BX Biofungicide, Hummerts) in individual 7.5 cm square pots (3” Kord) and were kept moist by misting for the first 7 days to aid germination. Seeds can also be germinated on agar-containing petri dishes and transplanted to soil once cotyledons emerge. Transplants were misted the first 2 days to acclimate to the growth chamber settings. Germination should occur within 7 days. Nitrogen (granulated prilled urea fertilizer, 46-0-0, 0.063 g/pot equivalent to 50 lbs. N /acre) was applied near the bottom of each pot at time of planting. Adding no fertilizer resulted in a shorter plant producing approximately 100-150 seeds. Flower buds were visible within 20-30 days after germination. A capillary mat watering system was used throughout plant growth to ensure plant uniformity and prevent leaching of nutrients, configured as follows. A 2 cm by 8 cm wick cut from Kimtech industrial wipes (Kimberly Clark 33352) was placed half way into the bottom of each pot. Pots were placed on a large piece of felt covering the lid of a plastic tub (Sterilite 32 quart) that was filled with water. Strips of white felt were placed into slots cut into the lid allowing water to wick from the tub to the large piece of felt. Plants finished growing and were ready for harvest after about 60 to 65 days. Increased rates of nitrogen stimulated tillering, branching, and total seed yield, while delaying harvest up to 10-15 days.

Growing pennycress in the field

For planting in the field, pennycress cultivars were drilled at a rate of 2.2 kg of seed per hectare in tilled soil at the Western Illinois University research farm in Macomb, Illinois. Drilled plots were established in September by a Hege 1200 drill/planter consisting of 9 rows, 7.5 cm apart at a depth of 0.3 cm (or just covered) into a fine, firm seed bed. Plot size measured 1 m by 3.5 m plots in a randomized split block design. All plots were fertilized with incorporated nitrogen (prilled urea) at a rate of 56 kg ha-1 and 11.2 kg ha-1 sulfur (Disper-Sul 90%). All plots were hand harvested in late May each year.

PCR Analyses

The following primer pair was used to amplify the *TaFAE1* gene in its entirety:
*Ta*FAE1_OuterF1: ACATGCATGTAAAACGTAACGG
*Ta*FAE1_OuterR1: TGGATTATATCAGGATGTGGCG
Product size: 1,842bp

FAE1 was amplified from genomic DNA of putative *fae1* CRISPR-Cas9 plants using Phusion DNA polymerase (NEB #M0530) with *Ta*FAE1_OuterF1 and *Ta*FAE1_OuterR1. Bands of the expected 1.8kb size were cut from a 1% agarose gel and DNA was extracted with the GeneJET Gel Extraction kit (#K0692) before being sequenced with the *Ta*FAE1_OuterF1 primer. DNA sequences were analyzed using either Benchling or Lasergene 13 software to identify/confirm the corresponding mutation.

Supporting Materials and Methods (cont.)

PCR analysis of transgenic plants

Genomic DNA was extracted from pennycress tissues using a standard CTAB extraction protocol described in Clarke et al., 2009. PCR was carried out using the following primers (or the *TaFAE1* primer pair listed above) to amplify portions of coding sequences from the noted genes of interest.

DsRed Forward: CCACAACACCGTGAAGCTGAAGG
DsRed Reverse: GGACTTGAACTCCACCAGGTAGTGG
Product size: 418bp

EaDAcT Forward: TTCATCAAGGTTTGGGTACAAGC
EaDAcT Reverse: AAGGAATTCAGCTAGGGTTGC
Product size: 528bp

Cas9 Forward: CACGCTCACGATGCTTACCTTA
Cas9 Reverse: ACCTTAGCCACAACGAGAACAGA
Product size: 500bp

Thermal cycling parameters were as follows: 1-minute initial denaturation at 98°C; 32 cycles of 98°C for 10 seconds, annealing at 55°C for 15 seconds, and a 72°C extension for 1 minute (*DsRed*, *EaDAcT* and *SpCas9*) or 2 minutes (*FAE1*); followed by a single 5-minute final extension at 72°C. Amplicons were detected by electrophoresis in a 1% agarose gel containing ethidium bromide and visualization with a UV transilluminator.

A

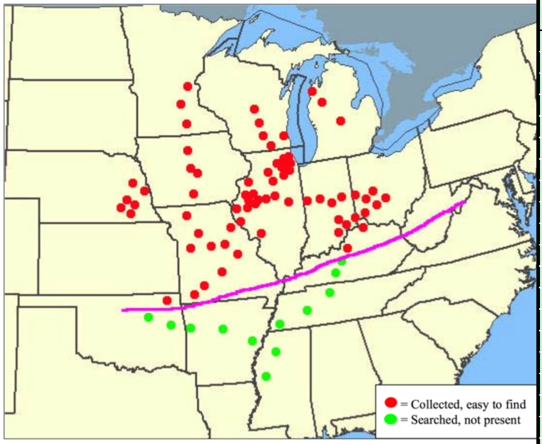

B

Sample Number	Collection Location	% Oil dwb	% Oleic Acid C18:1	% Linolenic Acid C18:3	% Erucic Acid C22:1	1000 sw (g)
11-1	Carthage, Illinois	30.51	12.37	11.83	32.39	0.79
11-2	Quincy, Illinois	31.26	7.72	11.54	32.82	0.94
11-3	Laddonia, Missouri	28.28	13.69	11.34	32.88	1.00
11-4	Holts Summit, Missouri	29.78	12.57	11.57	32.70	0.78
11-5	California, Missouri	26.81	11.78	11.30	32.23	1.00
11-6	Sedalia, Missouri	28.71	11.21	12.00	32.10	0.84
11-7	Carrollton, Missouri	27.45	15.46	10.19	30.21	0.89
11-8	Lincoln, Missouri	32.32	12.55	11.57	32.98	0.98
11-9	Lucas, Iowa	28.69	13.00	9.44	34.44	0.82
11-10	Ankeny, Iowa	27.35	12.00	10.08	37.02	0.88
11-11	Dows, Iowa	28.41	**17.13**	8.37	33.10	0.68
11-12	Glenville, Minnesota	25.14	13.51	9.66	34.83	0.65
11-13	Northfield, Minnesota	31.32	11.75	9.40	35.99	0.86
11-14	Harris, Minnesota	34.04	10.10	10.89	37.59	0.94
11-16	Oxford, Wisconsin	29.55	9.33	14.03	34.19	0.84
11-17	Vienna, Wisconsin	33.79	13.67	10.30	34.55	0.96
11-18	Janesville, Wisconsin	29.50	14.06	10.32	33.67	1.02
11-19	Rochelle, Illinois	29.75	12.15	10.28	36.57	1.14
11-20	Lydon, Illinois	28.90	11.50	10.15	35.31	0.94
11-21	Alexis, Illinois	26.93	12.51	11.82	32.76	1.24
11-22	Macomb, Illinois	30.13	13.38	10.27	31.53	0.90
11-23	Lincoln, Nebraska	24.47	15.05	9.32	30.52	0.67
11-24	Harvard, Nebraska	30.25	8.41	12.15	**38.41**	0.97
11-25	Mead, Nebraska	31.43	9.42	12.95	33.08	0.87
11-26	Lincoln, Nebraska	32.14	9.68	13.11	32.31	0.89
11-27	Omaha, Nebraska	27.10	11.07	10.68	35.38	0.79
11-28	Lincoln, Nebraska	30.37	10.48	12.48	34.13	0.98
Patton 1	Hanna City, Illinois	31.90	11.30	11.30	35.20	0.80
Beecher 2	Hanna City, Illinois	35.00	9.70	13.80	36.90	1.15
W1	McDonough County, Illinois	32.93	9.95	15.04	32.16	1.06
W2	McDonough County, Illinois	26.85	8.38	15.18	35.28	1.03
W3	McDonough County, Illinois	32.89	9.39	15.05	33.46	1.00
W4	McDonough County, Illinois	28.19	11.12	14.98	30.33	1.02
W5	McDonough County, Illinois	33.54	11.38	14.34	34.18	1.14
W6	McDonough County, Illinois	29.82	12.75	14.60	31.18	1.09
W7	McDonough County, Illinois	31.39	10.52	15.08	33.01	**1.34**
W8	McDonough County, Illinois	33.05	11.08	14.48	32.78	1.10
W9	McDonough County, Illinois	34.13	10.78	14.79	34.62	1.25
W10	McDonough County, Illinois	30.88	11.44	14.26	34.46	1.14
W11	McDonough County, Illinois	29.52	8.92	14.68	36.50	1.15
W12	McDonough County, Illinois	29.96	10.88	14.22	34.06	0.97
W13	McDonough County, Illinois	30.13	10.56	14.57	33.78	1.07
13-1	Roxana, Illinois	33.97	10.54	13.66	34.29	0.95
13-2	St. Clair, Missouri	31.70	12.40	13.68	31.43	0.94
13-3	Doolittle, Missouri	32.50	10.57	14.77	32.76	0.91
13-4	Phillipsburg, Missouri	32.67	10.60	14.00	32.30	0.85
13-5	Strafford , Missouri	31.15	12.70	14.33	28.49	0.94
13-6	Afton, Oklahoma	26.65	10.16	13.22	34.94	0.75
13-7	Hillview, Kentucky	30.81	10.38	14.03	33.55	0.96
13-8	Hanover, Indiana	35.00	11.83	14.37	29.78	0.90
13-9	Rexville, Indiana	33.16	10.45	14.20	31.43	0.84
13-10	Versailles, Indiana	33.16	11.86	13.72	31.84	0.79
13-11	Lawrenceburg, Indiana	31.51	11.60	15.07	30.02	0.84
13-12	Petersburg, Kentucky	28.81	8.59	**15.25**	35.36	0.84
13-13	Cleves, Ohio	32.04	11.57	14.08	31.54	0.86
13-14	Lebannon, Ohio	35.41	13.07	14.20	29.09	0.96
13-15	Wilmington, Ohio	31.30	13.75	14.93	27.46	0.96
13-16	Spring Valley, Ohio	26.23	9.87	12.71	33.79	0.70
13-17	Bath, Ohio	30.48	10.42	13.55	34.01	0.87
13-18	Lewisburg, Ohio	27.42	8.39	13.16	35.13	0.72
13-19	Newcastle, Indiana	32.21	12.74	12.42	29.91	0.83
13-20	Greenfield, Indiana	33.81	10.56	14.19	33.65	0.86
13-21	Lizton, Indiana	33.46	13.72	13.49	27.70	0.85
13-22	Covington, Indiana	34.82	10.10	14.09	33.91	0.83
13-23	Oakwood, Illinois	27.24	13.72	11.12	32.34	0.72
13-24	LeRoy, Illinois	28.44	8.79	14.92	36.58	0.81
13-25	San Jose, Illinois	27.71	11.20	13.11	37.28	0.85
13-26	Lewiston, Illinois	27.39	9.24	11.87	35.07	0.71
13-27	Adair, Illinois	24.43	11.87	13.48	31.02	0.57
13-28	Naperville, Illinois	34.74	9.19	13.86	35.24	0.86
13-29	Eola, Illinois	36.01	8.76	14.67	37.35	0.86
13-30	Aurora, Illinois	15.95	10.42	13.14	29.28	0.43
13-31	Metea Valley, Illinois	13.52	9.51	14.02	28.83	0.56
13-32	Oak Brook, Illinois	33.56	10.47	13.50	34.93	1.04
13-33	Batavia, Illinois	34.22	9.23	13.92	35.87	0.82
13-34	Geneva, Illinois	31.04	8.47	15.06	36.00	0.84
13-35	Hope, Michigan	**38.71**	9.27	13.10	35.80	1.01
13-36	Cadillac, Michigan	31.74	10.75	13.57	34.08	0.95
13-37	Frankfort, Michigan	32.00	9.60	13.83	34.67	1.01
13-38	Pleasant Prairie, Wisconsin	28.37	9.67	13.06	35.83	0.82
13-39	Rolling Meadows, Illinois	32.45	10.11	13.34	36.74	1.15
13-40	Macomb, Illinois	30.99	10.61	13.42	36.12	0.97
**Total Range**		13.52-38.71	7.72-17.13	8.37-15.25	27.46-38.41	0.43-1.34

Figure S1A, B. Collection locations and seed oil and weight characteristics for 80 wild pennycress populations collected throughout the U.S. Midwestern states. This represents a portion of the Western Illinois University collection. Note that pennycress plants were not readily found south of the Missouri and Kentucky borders. Numbers in red indicate highest levels recorded. The low seed oil content and weights of 13-30 and 13-31 were probably due to collection of immature seed. All samples are Winter type requiring vernalization. dwb = dry weight basis; 1000 sw = weight of 1,000 seeds in grams

Accession Number	Origin	Type	% Oil dwb	% Oleic Acid C18:1	% Linolenic Acid C18:3	% Erucic Acid C22:1	1000 sw (g)
Ames 22461	Poland	S	32.33	11.65	11.77	33.80	1.24
Ames 23761	Ontario, Canada	S	32.70	8.48	13.16	37.42	0.92
Ames 24499	Former Serbia	W	31.04	11.35	11.37	36.90	0.81
Ames 29118	Illinois, USA	W	27.64	9.17	13.45	37.14	0.79
Ames 29509	Ohio, USA	W	36.92	10.80	12.47	35.71	1.00
Ames 29512	Ohio, USA	W	27.30	8.64	12.46	37.94	0.60
Ames 29513	Iowa, USA	W	30.62	9.47	13.38	34.61	0.84
Ames 30982	Iowa, USA	W	32.44	9.53	14.26	34.89	0.83
Ames 30983	Illinois, USA	W	32.05	10.35	12.70	34.26	0.87
Ames 30984	South Dakota, USA	W	36.08	9.61	14.13	33.87	0.93
Ames 30985	South Dakota, USA	W	30.96	11.97	13.01	33.37	1.01
Ames 30997	Colorado, USA	W	24.73	**12.65**	12.14	29.81	0.76
Ames 30999	Colorado, USA	W	33.39	11.09	14.09	32.45	0.90
Ames 31012	Colorado, USA	W	34.04	9.13	14.68	32.78	0.81
Ames 31018-L1	Colorado, USA	W	33.96	11.36	13.83	32.52	0.92
Ames 31018-L2	Colorado, USA	W	32.05	11.30	13.02	35.20	**1.33**
Ames 31021	Colorado, USA	W	36.01	8.66	**14.98**	36.81	0.91
Ames 31023	Colorado, USA	W	34.46	9.28	13.99	36.88	0.96
Ames 31026	Colorado, USA	W	27.74	10.63	13.38	31.34	0.81
Ames 31487	Ontario, Canada	S	33.80	10.07	13.03	37.88	0.86
Ames 31488	Ontario, Canada	W	35.21	11.22	12.65	35.15	1.00
Ames 31489	Saskatchewan, Canada	S	36.09	11.32	13.12	34.74	1.04
Ames 31490	Saskatchewan, Canada	S	32.15	8.78	13.47	37.23	1.04
Ames 31491	Saskatchewan, Canada	S	31.26	8.70	12.73	36.45	0.92
Ames 31492	Saskatchewan, Canada	S	36.00	7.63	14.21	**38.95**	0.97
Ames 31493	Saskatchewan, Canada	S	34.54	9.49	12.98	36.97	1.18
Ames 31500	Alberta, Canada	S	**38.73**	8.29	13.12	37.68	1.14
Ames 31501	Manitoba, Canada	W	37.09	10.11	12.24	36.90	1.04
PI 633414	Thuringia, Germany	S	27.03	9.88	12.90	35.79	0.81
PI 633415	Saxony, Germany	S	25.86	9.22	10.33	36.52	0.72
PI 650284	Thuringia, Germany	S	31.56	10.03	8.90	35.86	0.84
PI 650285	Saxony, Germany	S	30.22	10.96	11.64	35.48	0.94
PI 650286	Saxony, Germany	S	26.88	10.65	9.09	35.96	0.81
PI 650287	Bas-Rhin, France	S	29.12	8.73	12.10	38.11	0.89
**Total Range**			24.73-38.73	7.63-12.65	8.90-14.98	29.81-38.95	0.60-1.33

Figure S2. Seed oil and weight characteristics of 34 USDA accessions of *Thlaspi arvense* L. (pennycress). Numbers in red indicate highest levels recorded. Type; W= Winter (requires vernalization), S= Spring. dwb = dry weight basis; 1000 sw = weight of 1,000 seeds in grams.

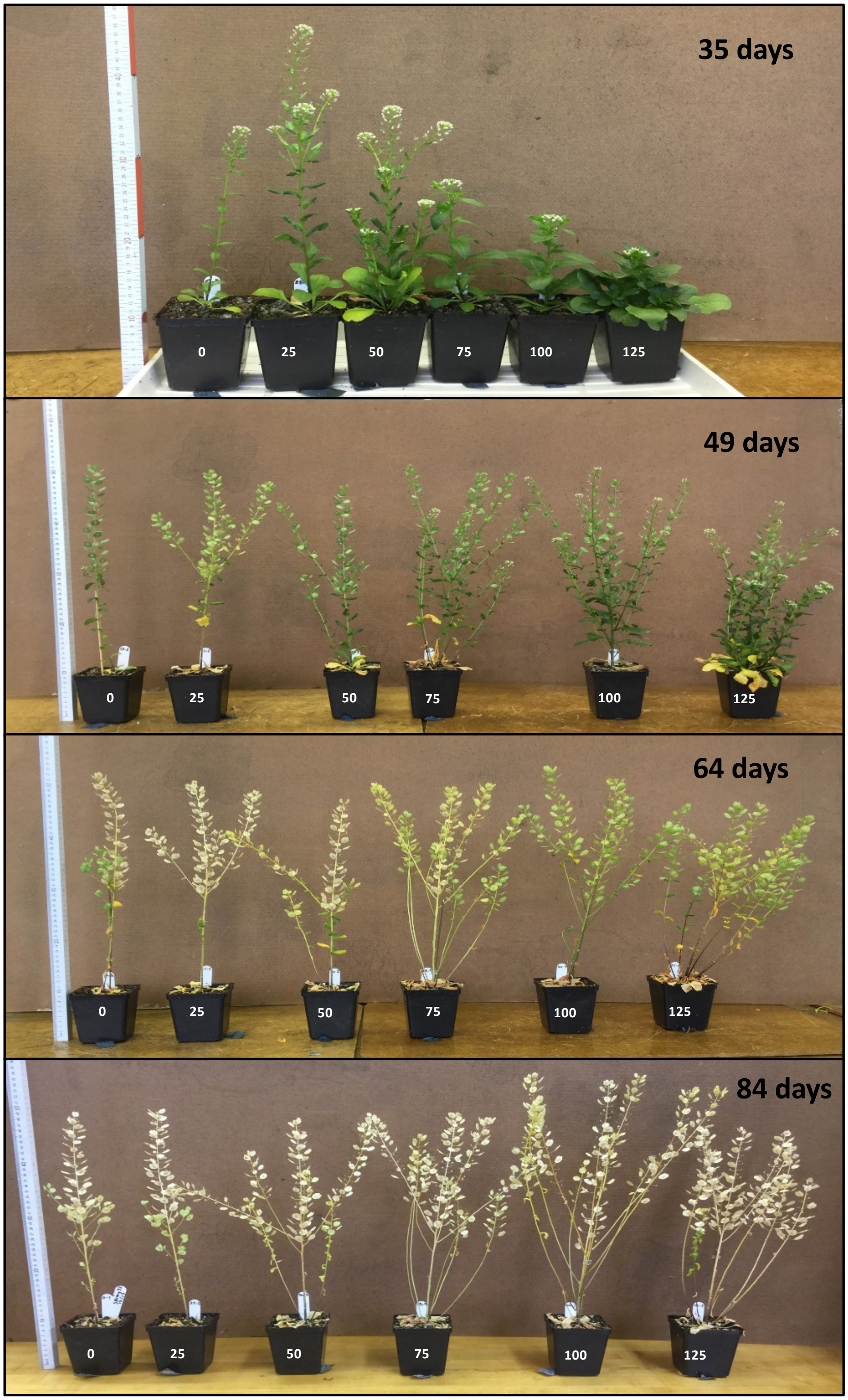

Figure S3. Development of pennycress cultivar Spring 32 plants in potting soil amended with five nitrogen (prilled urea) amounts, equivalent to 0, 25, 50, 75, 100, and 125 lbs N/acre. Plants were grown individually in 4-inch pots and pictures taken 35 to 84 days after planting. Note that plants grown with relatively low amounts of nitrogen flowered and senesced sooner and produced fewer seed pods.

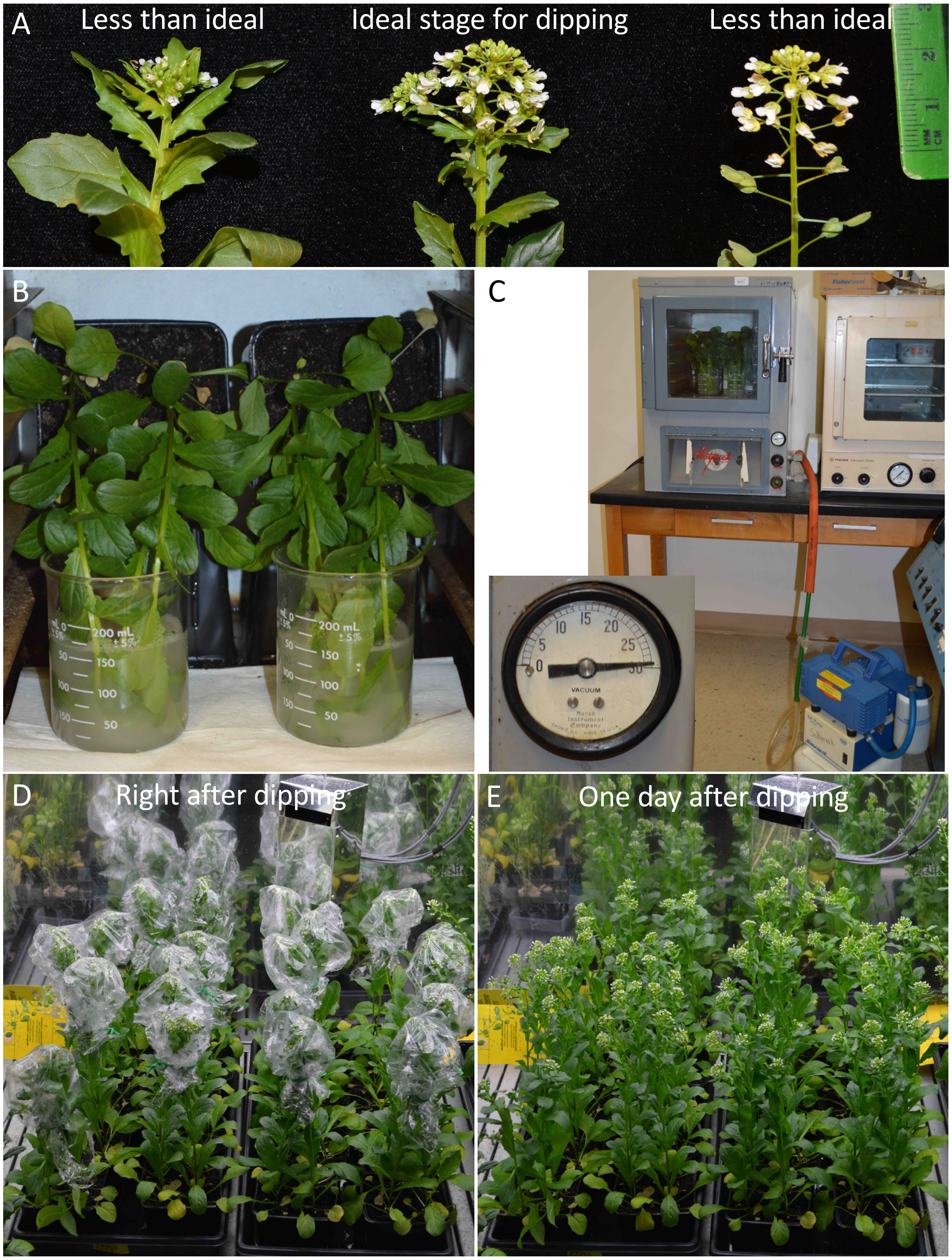

Figure S4. *Agrobacterium*-mediated floral transformation of pennycress. **A.** Pictured are three racemes of pennycress plants; Dipping of the raceme in the center is likely to produce the highest transformation efficiency. **B** and **C**. Racemes of plants submerged (dipped) in a solution containing *Agrobacterium tumefaciens (*strain GV3101), 5% sucrose, and 0.02% Silwet L-77, within a chamber put under 29 mm Hg (14.7 psi) vacuum. **D.** After the vacuum infiltration, the racemes were wrapped in plastic wrap sealed with twist ties, then placed in a growth chamber; **E.** Plastic wrap was removed the following day.

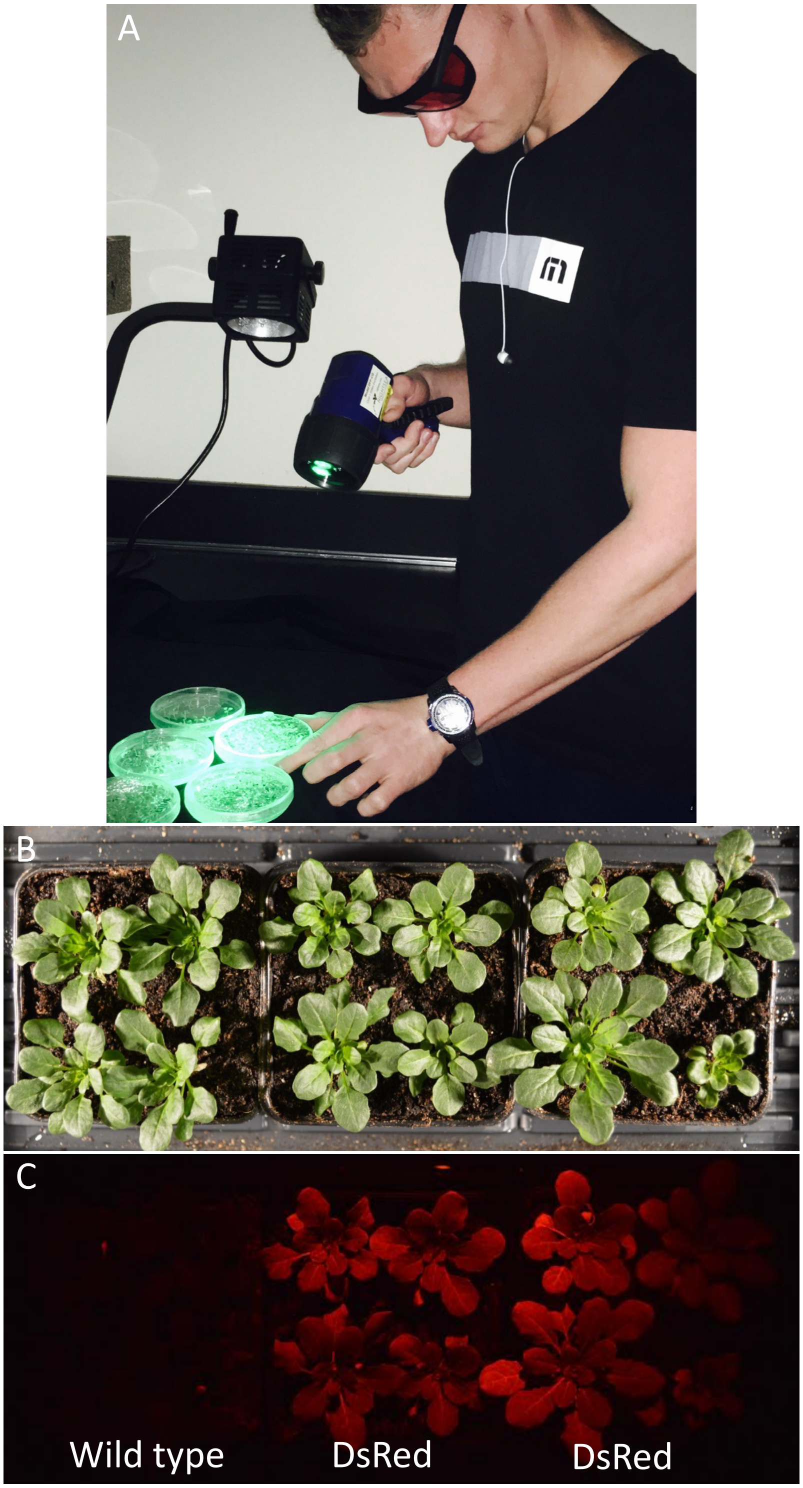

Figure S5A-C. Visualization of red fluorescence emanating from transgenic pennycress plants expressing the DsRed protein, using the NightSea fluorescent protein flashlight.

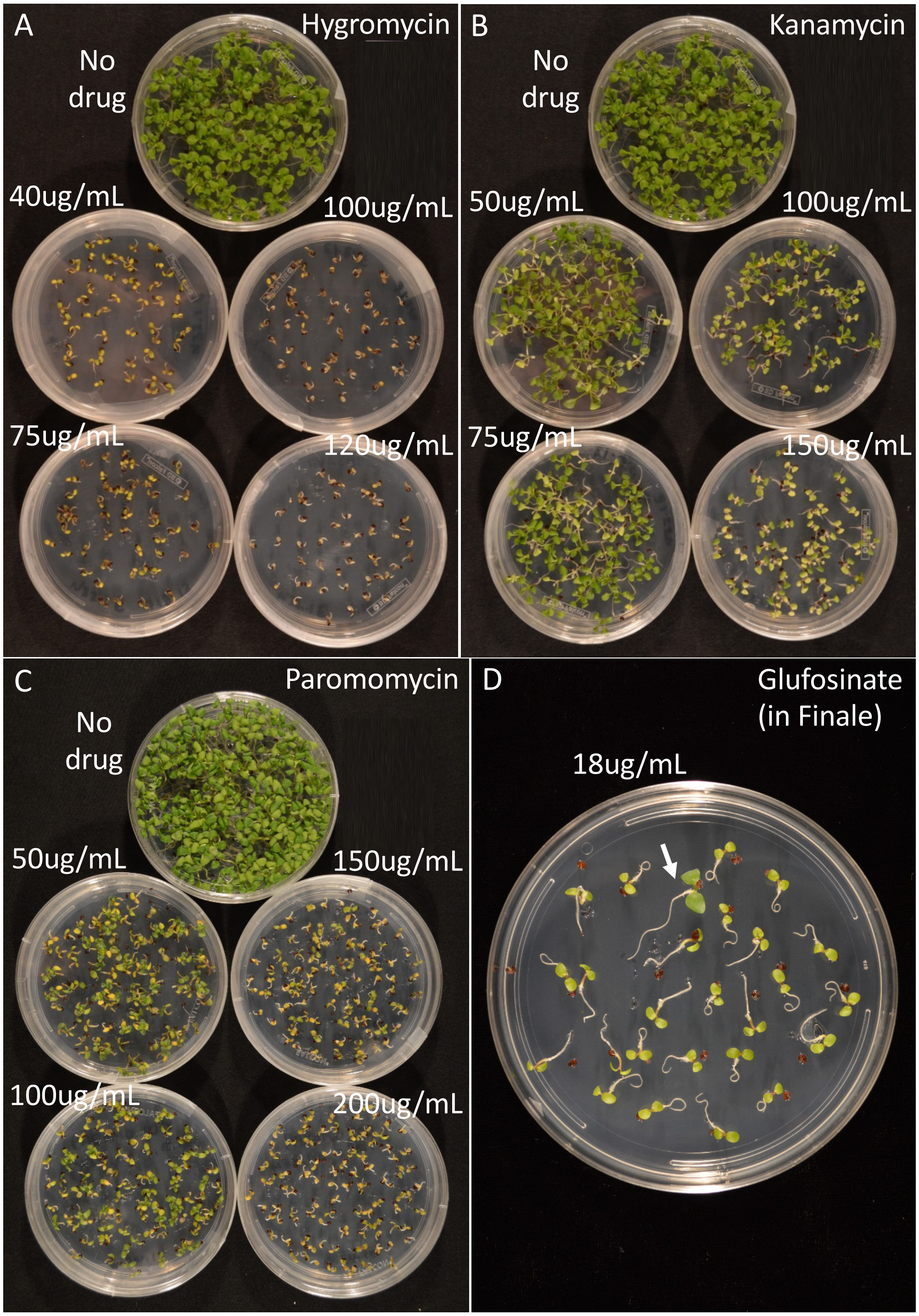

Figure S6A-D. Dose responses of untransformed pennycress seedlings germinated and grown on agar media containing various concentrations of **A.** hygromycin B, **B.** kanamycin, **C.** paromomycin, and **D.** glufosinate (in the herbicide Finale). Note that the glufosinate-containing plate (**D**) has one resistant seedling (delineated by an arrow) transgenic with the *Bar* gene.

Martinez/Needleman-Wunsch DNA Alignment
Minimum Match: 9; Gap Penalty: 1.10; Gap Length Penalty: 0.33

Seq1(1>1521) FAE1 ORF GAKE01018976	Seq2(1>1521) FAE1 ORF AT4G34520.seq	Similarity Index	Gap Number	Gap Length	Consensus Length
(1>1521)	(1>1521)	87.8	3	6	1524

```
         v10       v20       v30       v40       v50       v60       v70       v80       v90
ATGACGTCCGTTAACGTTAAGCTCCTTTACCATTACGTCATCACCAACTTTTTCAACCTTTGCTTCTTCCCGTTAGCGGCGATCGTTGCC
ATGACGTCCGTTAACGTTAAGCTCCTTTACC TTACGTC T ACCAACTTTTTCAACCT TG TT TTCCCGTTA CGGCG TC T GCC
ATGACGTCCGTTAACGTTAAGCTCCTTTACCGTTACGTCTTAACCAACTTTTTCAACCTCTGTTTGTTCCCGTTAACGGCGTTCCTCGCC
         ^10       ^20       ^30       ^40       ^50       ^60       ^70       ^80       ^90
         v100      v110      v120      v130      v140      v150      v160      v170      v180
GGAAAAGCCTCTCGGCTTACCACAAACGATCTTCACCACTTCTACTATTCCTATCTCCAACACAACCTAATAACCATATCTCTACTCTTT
GGAAAAGCCTCTCGGCTTACCA AAACGATCT CAC ACTTC  CT TTCCTATCTCCAACACAACCT ATAAC  TA CT TACTCTTT
GGAAAAGCCTCTCGGCTTACCATAAACGATCTCCACAACTTC--CT-TTCCTATCTCCAACACAACCTTATAACAGTAACTTTACTCTTT
         ^100      ^110      ^120      ^130         ^140      ^150      ^160      ^170
         v190      v200      v210      v220      v230      v240      v250      v260      v270
GCCTTCACCGTTTTCGGTTTGGCTCTCTACATCGTAACCCGGCCCAAACCGGTTTACCTCGTTGACCATTCCTGCTACCTTCCACCATCG
GC TTCAC GTTTTCGGTTTGG TCTCTACATCGTAACCCG CCCAA CCGGTTTA CTCGTTGAC A TC TG TACCTTCCACCA CG
GCTTTCACTGTTTTCGGTTTGGTTCTCTACATCGTAACCCGACCCAATCCGGTTTATCTCGTTGACTACTCGTGTTACCTTCCACCACCG
  ^180      ^190      ^200      ^210      ^220      ^230      ^240      ^250      ^260
         v280      v290      v300      v310      v320         v330      v340      v350
CATCTTAGAAGCAGTATCTCTAAGGTCATGGATATCTTCTATCAAGTAAGATTAGCCGA---TCCTTTACGGAACGCGGCAAGCGATGAT
CATCT A A   AGT TCTCTAA GTCATGGATAT TTCTA CAA TAAGA  AGC GA   T CTT ACGGAACG GGCA G GATGAT
CATCTCAAAGTTAGTGTCTCTAAAGTCATGGATATTTTCTACCAAATAAGAAAAGCTGATACTTCTTCACGGAACGTGGCATGTGATGAT
  ^270      ^280      ^290      ^300      ^310      ^320      ^330      ^340      ^350
  v360      v370      v380      v390      v400      v410      v420      v430      v440
TCGTCCTGGCTTGATTTCTTGAGGAAGATTCAGGAGCGGTCTGGTCTAGGCGATGAAACCCACGGCCCCGAGGGACTGCTTCAGGTCCCT
 CGTCCT GCT GATTTC TGAGGAAGATTCA GAGCG TC GGTCTAGG GATGA AC  AC G CC GAGGGACT  TTCA GT CC
CCGTCCTCGCTCGATTTCCTGAGGAAGATTCAAGAGCGTTCAGGTCTAGGTGATGAGACGTACAGTCCTGAGGGACTCATTCACGTACCA
  ^360      ^370      ^380      ^390      ^400      ^410      ^420      ^430      ^440
  v450      v460      v470      v480      v490      v500      v510      v520      v530
CCACGGAAGACTTTTGCCGCGGCGCGTGAAGAAACAGAGCAAGTGATCATCGGTGCGCTCGAAAAACTATTCGAGAACACCAAAGTTAAC
CC CGGAAGACTTTTGC GCG C CGTGAAGA ACAGAG A GT ATCATCGGTGCGCTCGAAAA CTATTCGAGAACACCAAAGTTAAC
CCGCGGAAGACTTTTGCAGCGTCACGTGAAGAGACAGAGAAGGTTATCATCGGTGCGCTCGAAAATCTATTCGAGAACACCAAAGTTAAC
  ^450      ^460      ^470      ^480      ^490      ^500      ^510      ^520      ^530
  v540      v550      v560      v570      v580      v590      v600      v610      v620
CCTAAAGAGATTGGTATACTTGTGGTGAACTCAAGCATGTTTAATCCGACTCCTTCGCTCTCGGCGATGGTTGTTAATACTTTCAAGCTC
CCTA AGAGATTGGTATACTTGTGGTGAACTCAAGCATGTTTAATCC ACTCCTTCGCT TC GC ATGGT GTTAATACTTTCAAGCTC
CCTAGAGAGATTGGTATACTTGTGGTGAACTCAAGCATGTTTAATCCAACTCCTTCGCTATCCGCTATGGTCGTTAATACTTTCAAGCTC
  ^540      ^550      ^560      ^570      ^580      ^590      ^600      ^610      ^620
  v630      v640      v650      v660      v670      v680      v690      v700      v710
CGAAGCAACATCAGAAGCTTTAATCTTGGAGGAATGGGTTGTAGTGCCGGCGTTATAGCCATTGATCTGGCTAAGGACTTGTTGCATGTC
CGAAGCAACATCA AAGCTTTAATCT GGAGGAATGGGTTGTAGTGC GG GTTAT GCCATTGAT TGGCTAA GACTTGTTGCATGT
CGAAGCAACATCAAAAGCTTTAATCTAGGAGGAATGGGTTGTAGTGCTGGTGTTATTGCCATTGATTTGGCTAAAGACTTGTTGCATGTT
  ^630      ^640      ^650      ^660      ^670      ^680      ^690      ^700      ^710
  v720      v730      v740      v750      v760      v770      v780      v790      v800
CATAAAAACACTTATGCTCTTGTGGTGAGCACAGAGAACATCACTTACAACATTTATGCTGGTGATAACAGATCCATGATGGTTTCGAAT
CATAAAAACACTTATGCTCTTGTGGTGAGCAC GAGAACATCAC  A   CATTTATGCTGG GA AA AGATC ATGATGGTT   AAT
CATAAAAACACTTATGCTCTTGTGGTGAGCACTGAGAACATCACACAAGGCATTTATGCTGGAGAAAATAGATCAATGATGGTTAGCAAT
  ^720      ^730      ^740      ^750      ^760      ^770      ^780      ^790      ^800
  v810      v820      v830      v840      v850      v860      v870      v880      v890
TGCTTGTTCCGTGTTGGTGGGGCCGCGATTTTGCTCTCCAACAAGCCGAGGGACCGGAGACGGTCCAAGTACCAGCTACTTCACACGGTT
TGCTTGTT CGTGTTGGTGGGGCCGCGATTTTGCTCTC AACAAG CG G GACCGGAGACGGTCCAAGTAC AGCTA TTCACACGGT
TGCTTGTTTCGTGTTGGTGGGGCCGCGATTTTGCTCTCTAACAAGTCGGGAGACCGGAGACGGTCCAAGTACAAGCTAGTTCACACGGTC
  ^810      ^820      ^830      ^840      ^850      ^860      ^870      ^880      ^890
  v900      v910      v920      v930      v940      v950      v960      v970      v980
CGGACGCATACCGGAGCTGACGACAAGTCTTTCCGATGTGTGCAACAAGAAGACGACGAGAGCGGTAAAACCGGGGTGTGTTTGTCCAAG
CG ACGCATAC GGAGCTGA GACAAGTCTTT CGATGTGTGCAACAAGAAGACGA GAGAGCGG AAAA CGG GT TGT TGTC AAG
CGAACGCATACTGGAGCTGATGACAAGTCTTTTCGATGTGTGCAACAAGAAGACGATGAGAGCGGCAAAATCGGAGTTTGTCTGTCAAAG
  ^900      ^910      ^920      ^930      ^940      ^950      ^960      ^970      ^980
  v990      v1000     v1010     v1020     v1030     v1040     v1050     v1060     v1070
GACATAACCGGTGTTGCCGGGAGAACTGTTCAGAAAAACATAACAACATTGGGTCCGTTGGTTCTTCCTTTTAGCGAGAAATTTCTTTTT
GACATAACC  TGTTGC GGGA AAC  TT  GAAAAA ATA CAACATTGGGTCCGTTG TTCTTCCTTT AGCGA AA TTTCTTTTT
GACATAACCAATGTTGCGGGGACAACACTTACGAAAAATATAGCAACATTGGGTCCGTTGATTCTTCCTTTAAGCGAAAAGTTTCTTTTT
  ^990      ^1000     ^1010     ^1020     ^1030     ^1040     ^1050     ^1060     ^1070
  v1080     v1090     v1100     v1110     v1120     v1130     v1140     v1150     v1160
TTCGTTACCTTCATCGCCAAGAAACTCTTTAAAGACAAGATCAAACATTACTACGTCCCGGATTTCAAGCTTGCTATCGACCATTTTTGT
TTCG TACCTTC TCGCCAAGAAACT  T AA GA AA ATCAA CATTACTA GT CCGGATTTCAAGCTTGCT T GACCATTT TGT
TTCGCTACCTTCGTCGCCAAGAAACTTCTAAAGGATAAAATCAAGCATTACTATGTTCCGGATTTCAAGCTTGCTGTTGACCATTTCTGT
  ^1080     ^1090     ^1100     ^1110     ^1120     ^1130     ^1140     ^1150     ^1160
  v1170     v1180     v1190     v1200     v1210     v1220     v1230     v1240     v1250
ATTCATGCCGGAGGCAGAGCCGTGATCGATGTGCTACAGAAGAACTTAGGTCTATTGCCGATCGATGTGGAGGCATCTAGGTCAACGTTA
ATTCATGCCGGAGGCAGAGCCGTGATCGATG GCTA AGAAGAACTTAGG CTAT GCCGATCGATGTGGAGGCATCTAG TCAACGTTA
ATTCATGCCGGAGGCAGAGCCGTGATCGATGAGCTAGAGAAGAACTTAGGACTATCGCCGATCGATGTGGAGGCATCTAGATCAACGTTA
  ^1170     ^1180     ^1190     ^1200     ^1210     ^1220     ^1230     ^1240     ^1250
  v1260     v1270     v1280     v1290     v1300     v1310     v1320     v1330     v1340
CATAGATTTGGGAACACTTCGTCTAGCTCAATTTGGTATGAATTGGCGTACATAGAGGCAAAAGGAAGGATGAAGAGAGGGAACAAAGTT
CATAGATTTGGGAA ACTTC TCTAGCTCAATTTGGTATGAATT GC TACATAGAGGCAAA GGAAG ATGAAGA AGGGAA AAAG T
CATAGATTTGGGAATACTTCATCTAGCTCAATTTGGTATGAATTAGCATACATAGAGGCAAAGGGAAGAATGAAGAAAGGGAATAAAGCT
  ^1260     ^1270     ^1280     ^1290     ^1300     ^1310     ^1320     ^1330     ^1340
```

Figure S7. Nucleotide sequence alignment of the *Arabidopsis thaliana AtFAE1* gene (At4g34520) ORF and the *Thlaspi arvense TaFAE1* ORF (derived from transcriptome assembly contig GAKE01018976; Dorn et al., 2014). The red line delineates the location of the 20 bp protospacer used in the CRISPR-Cas9 construct; the red box delineates the NGG protospacer-adjacent motif (PAM) site.

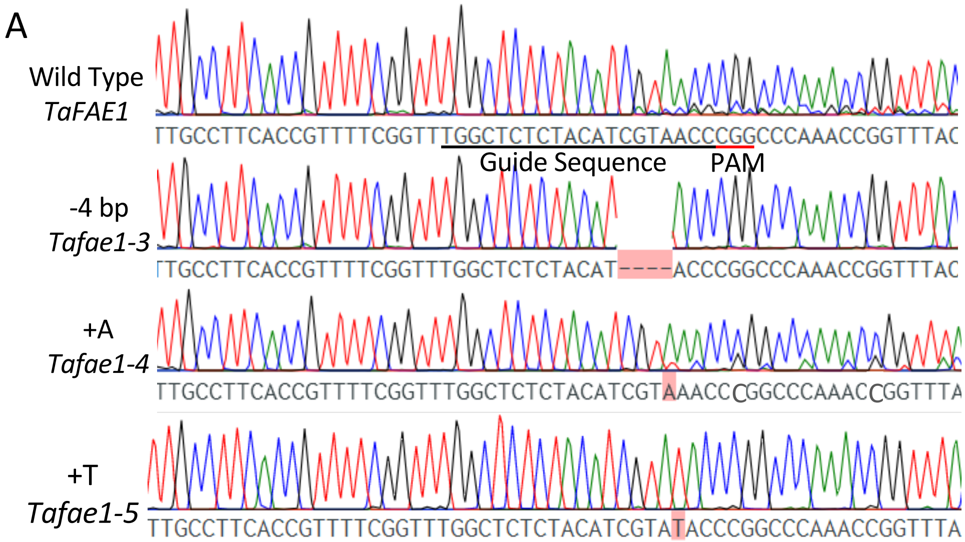

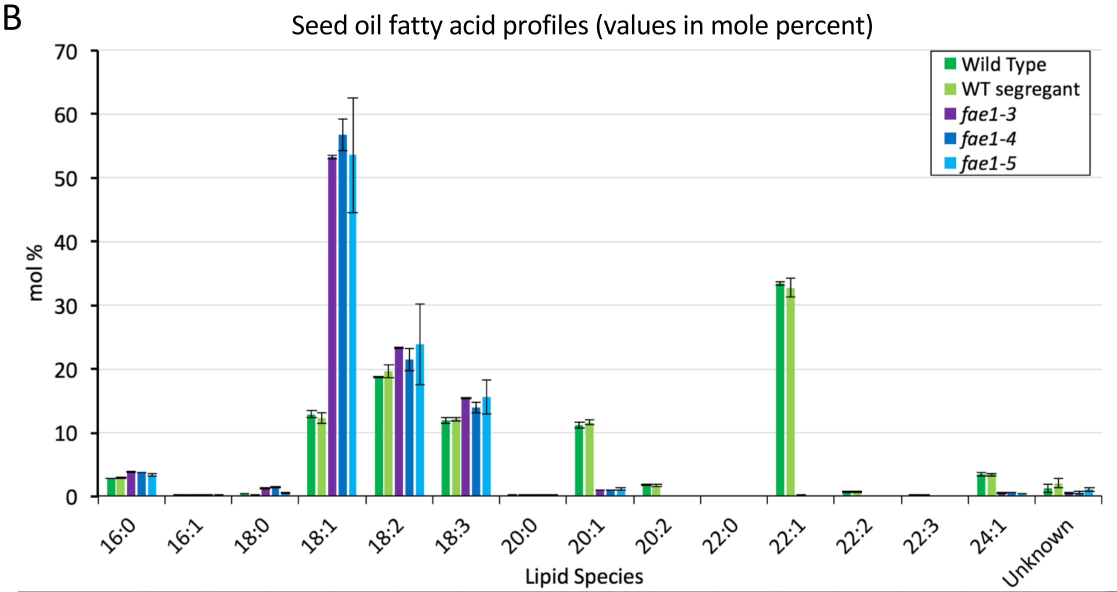

	16:0	16:1	18:0	18:1	18:2	18:3	20:0	20:1	20:2	22:0	22:1	22:2	22:3	24:1	unknown
**Wild Type**	2.7	0.2	0.4	13.4	16.5	9.0	0.2	15.0	1.6	0.0	35.3	0.6	0.2	3.2	1.0
**WT segregant**	2.8	0.3	0.4	10.2	18.6	14.0	0.2	10.8	1.9	0.1	34.4	0.8	0.3	3.6	1.0
fae1-3	3.9	0.3	1.2	53.3	23.4	15.4	0.3	0.9	0.1	0.0	0.2	0.0	0.0	0.5	0.6
fae1-4	3.5	0.2	0.9	52.1	25.1	16.1	0.2	0.9	0.0	0.0	0.0	0.0	0.0	0.4	0.5
fae1-5	3.3	0.2	0.6	54.7	22.6	15.2	0.1	1.2	0.1	0.0	0.1	0.0	0.0	0.4	1.4

Figure S8A, B. DNA sequence chromatograms showing the CRISPR-Cas9 induced deletion and insertion mutations in the *fae1-3*, *fae1-4*, and *fae1-5* mutants along with the location of the CRISPR guide sequence and PAM site. The allele name and nature of each mutation is listed to the left of the chromatogram. These mutations were found to be heritable in T_3 and T_4 generation plants and co-segregate with the soil oil fatty acid profile phenotypes shown in (**B**).

Arabidopsis Root tip

Methylene Blue stain , 40X magnification

Pennycress Root tip

Methylene Blue stain , 40X magnification

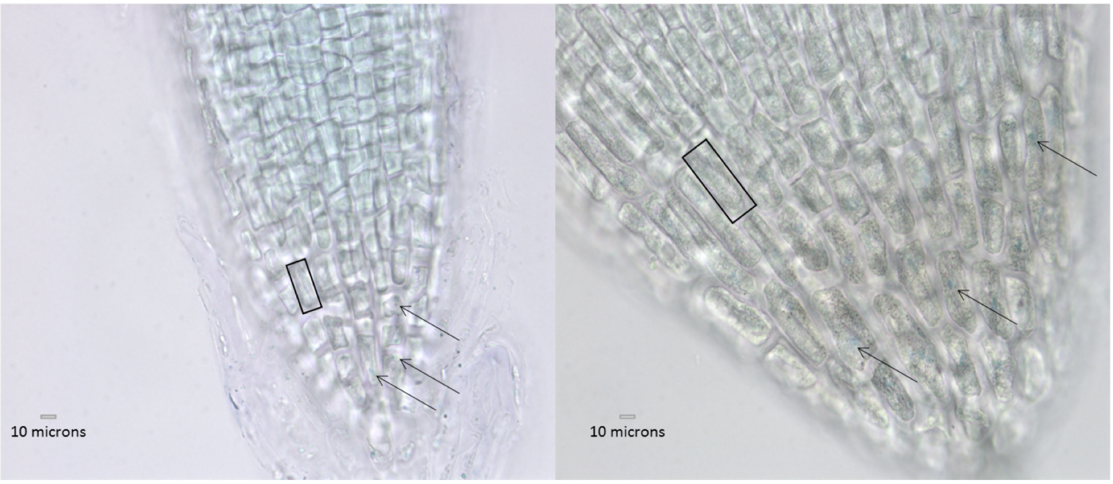

Figure S9. Microscopic images comparing the cell sizes (boxes) and nuclei (arrows) in Arabidopsis versus pennycress roots stained with methylene blue for visualization. Images are shown at the same scale.
